# Supplementary material for: Exo-selective intermolecular Diels–Alder reaction by PyrI4 and AbnU on non-natural substrates
Source: Commun Chem. 2021 Aug 3;4:113. doi: 10.1038/s42004-021-00552-9 (PMC9814550; doi:10.1038/s42004-021-00552-9)
Supplement: Supplementary file 1 — Supplementary Information [file 42004_2021_552_MOESM1_ESM.pdf]

Supplementary Information

Exo-selective intermolecular Diels-Alder reaction by PyrI4 and AbnU on non-natural substrates

Rajnandani Kashyap[1,2], Naga Veera Yerra[2,3], Joachyutharayalu Oja[4], Sandeepchowdary Bala[1,2], Gal Reddy Potuganti[2,4], Jagadeshwar Reddy Thota[2,3], Manjula Alla[2,4], Debnath Pal[5] and Anthony Addlagatta[1,2]*

[1] Department of Applied Biology, CSIR-Indian Institute of Chemical Technology, Uppal Road, Hyderabad, 500007, Telangana State, India.

[2] Academy of Scientific and Innovative Research (AcSIR), Ghaziabad - 201002, India

[3] Analytical and Structural Chemistry Department, CSIR-Indian Institute of Chemical Technology, Uppal Road, Hyderabad, 500007, Telangana State, India.

[4] Fluoro-Agrochemicals Department, CSIR-Indian Institute of Chemical Technology, Uppal Road, Hyderabad, 500007, Telangana State, India.

[5] Department of Computational and Data Sciences, Indian Institute of Science, Bengaluru, India.

*To whom correspondence should be addressed:
Dr. Anthony Addlagatta, Department of Applied Biology, CSIR – Indian Institute of Chemical Technology, Uppal Road, Tarnaka, Hyderabad – 500007, Telangana, India. Tel. +91-9493402208, Email: anthony@csiriict.in

Supplementary Methods and Results

Materials: All chemicals required for experiments were purchased from Merck (Sigma-Aldrich, USA). All molecular biology enzymes were purchased from New England Biolabs, USA. Details about the equipment, clones, reagents, columns etc. used in this study are referred in experimental part as and when required.

1. Sequence and structural analyses the β-barrel proteins

Structures of some β-barrel proteins known for their Diels-Alderase or cyclase activity have been retrieved from the PDB[1] and aligned in Pymol.[2] They share though very low sequence similarity but share high structural homology (Supplementary Figure 1). Sequence of PyrI4, AbyU and AbnU were aligned to understand the sequence identity between three homologues (Supplementary Figure 2).

Supplementary Figure 1: Structures of representative β-barrel proteins that carryout Diels-Alder reactions. PDB ID's are given in the brackets.

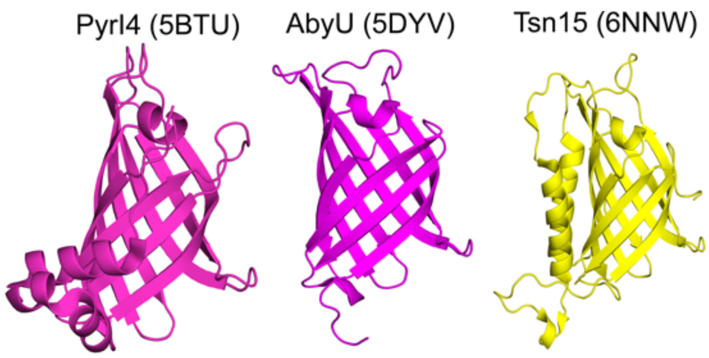

Supplementary Figure 2: Sequence alignment of AbnU with its closest homologue AbyU (PDB: 5DYV) and PyrI4 (PDB: 5BU3) depicting the conserved residues generated by Jalview 2.10.5 software.[3] The associated NCBI accession codes and their respective sequence identity to AbnU are also shown for both the homologs.

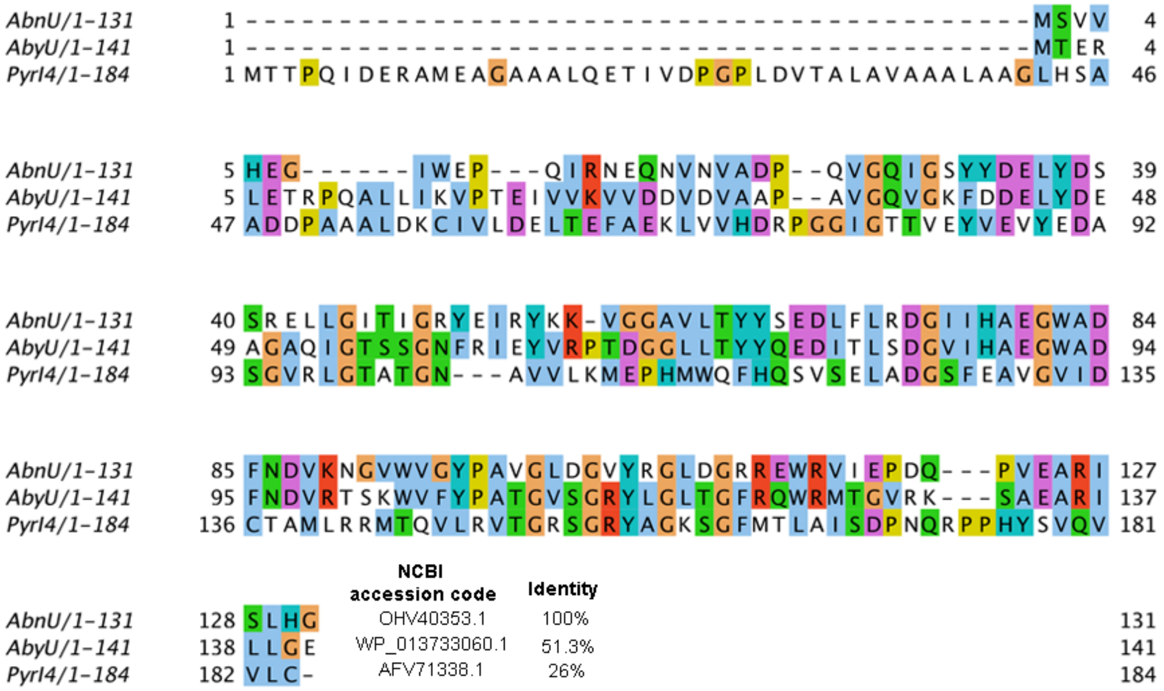

2. Gene Cloning

Genes encoding PyrI4, PyrI4-Δ10 (both from *Streptomyces rugosporus*) and AbnU (NCBI sequence id: WP_157905384.1, *Frankia sp. Cc1.17,* 393 *bp*) were codon optimized and synthesized by Genscript USA in pET28a (+) vector with N-terminal polyhistidine-tag. PyrI4 and PyrI4-Δ10 were inserted between restriction sites NdeI and HindIII. AbnU was inserted between restriction sites NdeI and XhoI. Single-point mutations were introduced by using site-directed mutagenesis strategy utilizing PCR-based methods followed by *Dpn*I (New England Biolabs Inc.) digestion, transformation into Dh5α *E. coli* strain, and screening of positive clones by nucleotide sequencing. All primers used for site-directed mutagenesis are summarized in the table below (Supplementary Table 1).

Supplementary Table 1: DNA Primers used for site-directed mutagenesis of PyrI4 in this study.

Primer	Sequence
PyrI4-E65A-for	GAGCTGACCGCGTTCGCGGAGAAACTGGTGGTTCAC
PyrI4-E65A-rev	CTCCGCGAACGCGGTCAGCTCATCCAGCACAATGC

PyrI4-E65Q-for	GAGCTGACCCAATTCGCGGAGAAACTGGTGGTTCAC
PyrI4-E65Q-rev	CTCCGCGAATTGGGTCAGCTCATCCAGCACAATGC
PyrI4-Δ10-Q115E-for	CACATGTGGGAGTTCCACCAAAGCGTTAGCGAGC
PyrI4-Δ10-Q115E-rev	TTGGTGGAACTCCCACATGTGCGGCTCCATCTTC

3. Protein expression

All clones (AbnU, PyrI4, PyrI4-Δ10 and all mutants) were transformed into *Escherichia coli* BL21 (DE3). Cultures of transformed *E. coli* BL21 (DE3) cells were grown in 1 L Luria-Bertani (LB) medium, supplemented with 50 μg/mL kanamycin, at 37 °C with shaking, until the optical density of the cultures at 600 nm had reached 0.8. Protein expression was induced by the addition of IPTG to a final concentration of 0.5 mM. Cultures were grown for an additional 12 h with shaking at 37 °C, following which the cells were harvested by centrifugation and the cell pellet stored at −80 °C for further purification using Ni-NTA resin with a standard affinity purification protocol (as mentioned below).

4. **Protein purification and characterization**

All recombinant proteins were purified using the same general strategy. For purification, harvested cells were thawed and suspended in the lysis buffer A (50 mM Tris, 200 mM NaCl [pH 8.0] supplemented with 1 mM PMSF). Bacterial cells were lysed by sonication (pulse of 1 sec ON, 3 sec OFF) and then centrifuged (SS-34 rotor) at 38,724 *g* for 40 minutes at 4 °C. The supernatant was loaded on to a 5 mL His Ni-NTA column (Sigma). The column was equilibrated with binding buffer A prior to loading the supernatant. Washed the column with buffer A to remove impurities and eluted with a step gradient of 2%, 5%, 10%, 20%, 50%, and 100% of buffer B (50 mM Tris, 200 mM NaCl [pH 8.0], 500 mM imidazole, pH 8.0). Fractions found to contain the target protein of interest, as determined by monitoring the absorbance of the column eluent at 280 nm followed by SDS-PAGE analysis, were dialyzed against the buffer C (20 mM Tris [pH 8.0], 100 mM NaCl) to remove imidazole. Dialyzed proteins were concentrated to at least 10 mg/ml. The concentration was estimated by Bradford assay and flash-frozen in liquid nitrogen for storage at -80 °C. PyrI4 protein was stored with 1mM DTT and 1mM EDTA (for crystallization purpose only). Pure concentrated proteins were analyzed on SDS-PAGE for purity (>95% pure) (Supplementary Figure 3a-b).

Size exclusion chromatography: To characterize the oligomer state of AbnU in solution, we performed size exclusion chromatography (SEC) using the Bio-Rad NGC chromatography system. S200 column (XK-16/70 (GE Life Sciences) packed manually) was pre-calibrated with blue dextran

for the void volume and a calibration standard (Bio-Rad gel filtration standard, cat: 1511901) were run for standard plot as shown in Supplementary Figure 3c. Concentrated AbnU protein (20 mg/ml) was spinned and loaded onto a pre-equilibrated S200 column with buffer E (20 mM Tris, [pH 8.0], 100 mM NaCl). SEC fractions were eluted, monitored at 280 nm (multi wavelength detector; an inbuilt system feature) and analyzed on SDS gel (Supplementary Figure 3c) for purity and monomer mass. Mass of AbnU, PyrI4 and PyrI4-Δ10 is determined by MALDI-reTOF/TOF UltrafleXtreme (Bruker Daltonics, Bremen, Germany) (Supplementary Figure 3d).

Thermal shift assay: 20 μM of protein (AbnU, PyrI4, PyrI4-Δ10 and mutants) in buffer D (20 mM Tris [pH 8.0] and 50 mM NaCl) and 6.25x SYPRO Orange dye (5000x stock concentration in DMSO, Sigma) reaction was setup to a volume of 20 μl in a 96-well PCR plate. PCR plate was centrifuged at 200 x g for 5 minutes before monitoring the change in SYPRO Orange fluorescence by real-time PCR instrument (Biorad CFX96 Real-Time System). Fluorescence was monitored with a temperature scan from 4 °C to 95 °C with an increment of 1 °C /min. The differential of the fluorescence intensity (dF/dT) versus temperature plot gave an estimate of melting temperature (T_m) of all the individual proteins and hence their thermal stability (Supplementary Figure 3e).

Supplementary Figure 3: SDS-PAGE gel analysis of purified recombinant proteins PyrI4 and its mutant: a) PyrI4 and its mutant protein with a protein standard marker (**M**) on 12% SDS-PAGE gel b) Comparative gel (15% SDS-PAGE gel) of purified PyrI4 and PyrI4-Δ10 mutant showing a difference of about 1.0 kDa corresponding to the lack of N-terminal 10 residues in PyrI4-Δ10.

a)

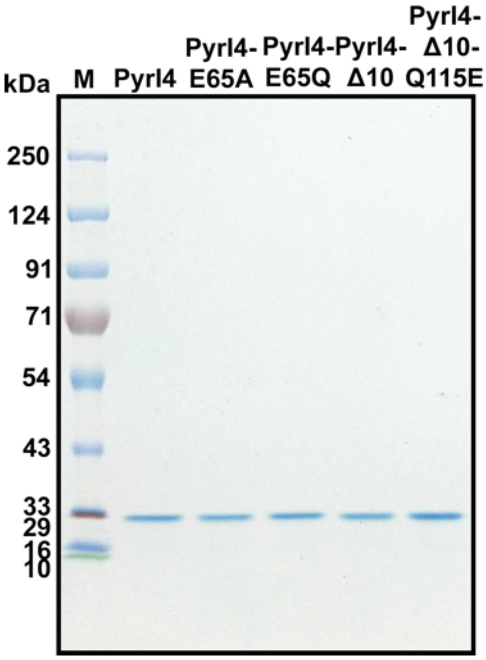

b)

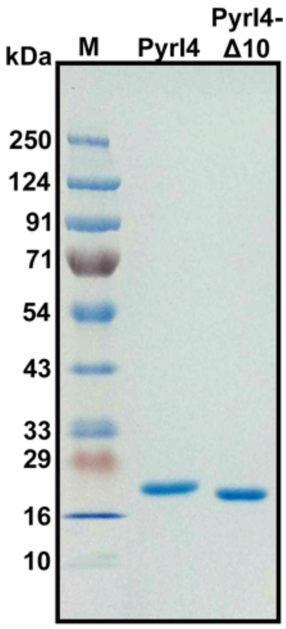

c) Size exclusion chromatogram and SDS-PAGE gel analysis of the purified recombinant protein AbnU with the standard gel filtration standard plot.

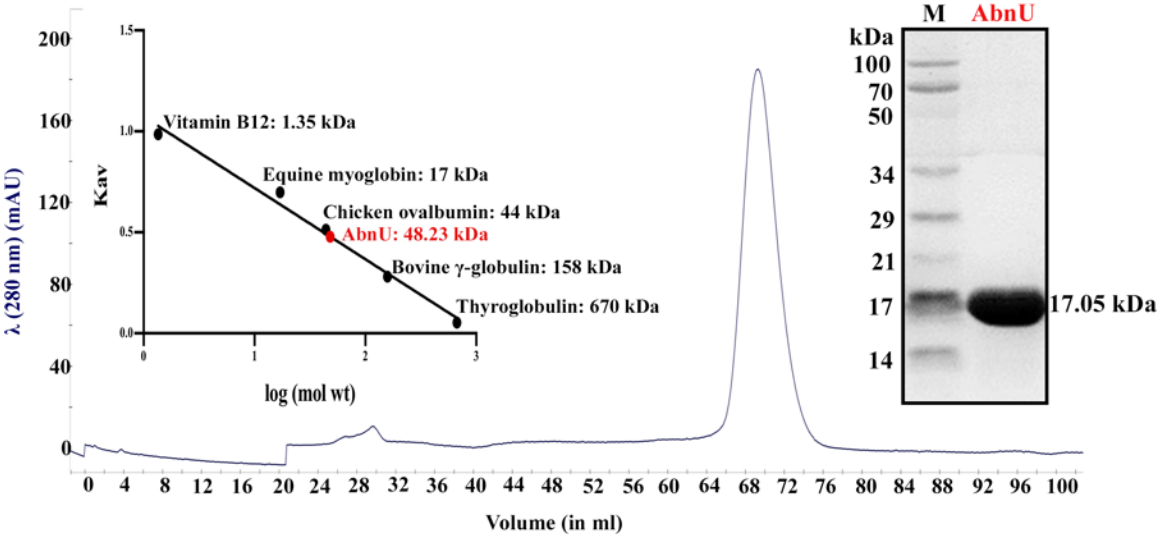

d) Positive mode MALDI-reTOF/TOF (linear mode) mass spectra of A) AbnU, B) PyrI4, and C) PyrI4-Δ10 using 2-Cyano-3-(2-thienyl)acrylic Acid (CTA) as matrix [4]

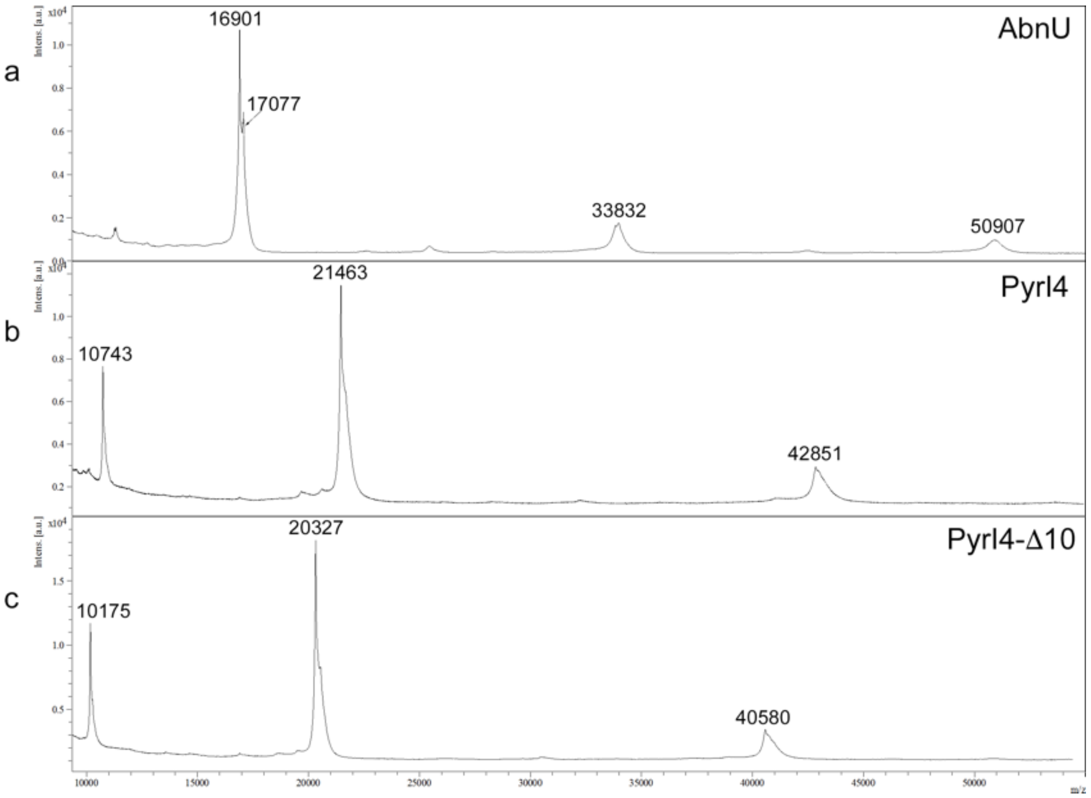

e) Thermal shift assay for AbnU, PyrI4 and its mutants depicted by plot of differential of the fluorescence intensity (dF/dT) versus temperature plot. Corresponding T_m are included in °C.

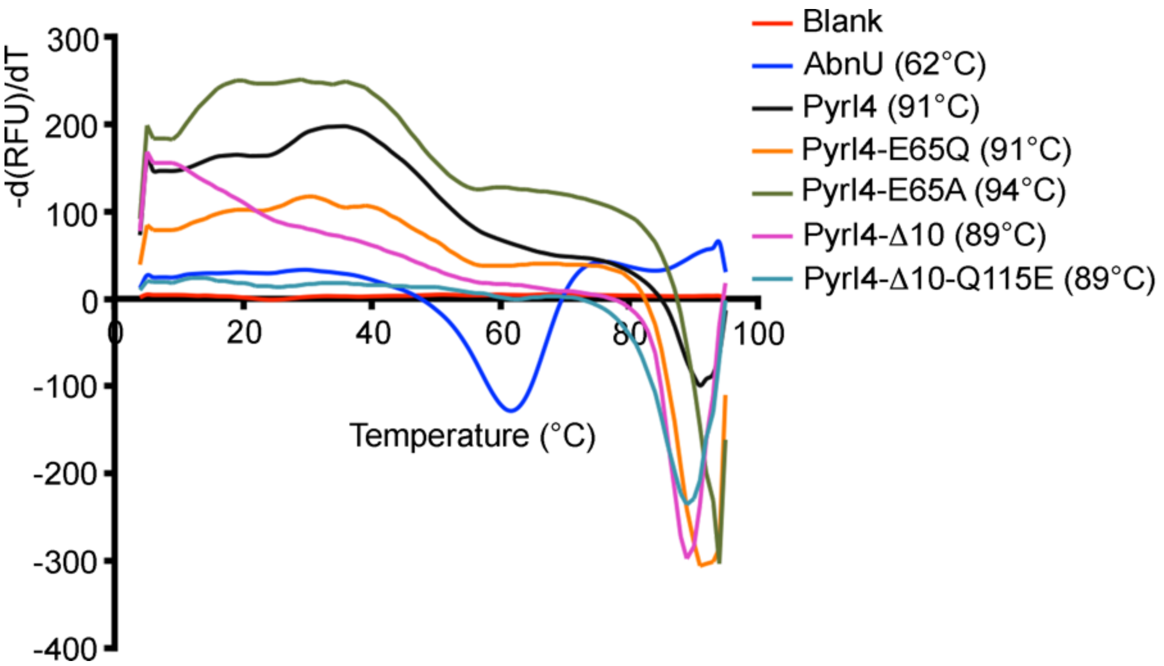

5. Protein Crystallization

The initial attempts failed to crystallize the PyrI4 full length protein using published conditions.[5] We screened all recombinant proteins, namely AbnU, PyrI4 and PyrI4-Δ10 using 12 different crystallization conditions (Hampton Research and Molecular Dimensions), which correspond to about 1200 reagents. 10 mg/ml of protein was used to set up the crystallization screen by mixing 0.2 μl of protein and 0.2 μl of reservoir solution via the sitting drop vapor diffusion method. Crystal trays were kept at 25 °C in a temperature-controlled and vibration free cabinet.

Crystallization conditions for all proteins were identified. Diffraction quality crystals were obtained by further optimization of the hit conditions with the hanging drop vapor diffusion method at 25°C. Diffraction quality co-crystals for PyrI4 were grown in reservoir solution comprising of reservoir solutions 0.02 M calcium acetate hydrate, 0.1 M sodium cacodylate pH 6.5, 42% v/v PEG 300 in the presence of 1 mM **3a**. Reservoir for PyrI4-Δ10 comprised of 6% Polypropylene glycol P400 and 2% glycerol and 1 mM **3a**. Reservoir buffer for AbnU protein contained 1M Imidazole, pH 7.0 and 20% ethanol. Diffraction quality crystal appeared in 24-48 hrs. Both co-crystallization and soaking techniques were employed to determine the ligand bound crystals.

6. Optimization of the chromatographic method and Mass spectrometry

a) Quantitative study:

Molecular separation was carried out using a UHPLC system (Ultimate 3000, Thermo Scientific, Bremen, Germany) equipped with a quaternary pump, an in-line degasser, and an auto-injector and column compartment. The reversed-phase chromatographic separations were performed on a Hypersil Gold C18 (Dimensions 100 x 2.1 (mm), 1.9 (μ) particle size, Thermo scientific) using 0.1 % formic acid in water (A) as buffer and acetonitrile (B) as organic modifier for gradient elution. The flow rate was 0.5 mL/min., the column compartment temperature was maintained at 25 °C, the autosampler was kept at 7 °C and the injection volume was 10 μl. The elution gradient was optimized at: (time in min/ % of solvent B): 0-0.3/5, 0.3-4/95, 4-4.6/95, 4.6-4.7/5, and 4.7-5.5/5. The column was equilibrated for 2 min with an initial gradient before the next injection and three independent measurements were taken.

The eluent from the UHPLC column was directed into the mass spectrometer (TSQ Altis, Thermo Scientific, Bremen, Germany) through heated electrospray ionization (HESI) interface operated in the positive ion mode. The interface conditions were as follows: vaporizer temperature 350 °C, ion transfer tube temperature 325 °C, sheath gas flow 40 (arbitrary units), auxiliary gas flow gas flow 10 (arbitrary units), sweep gas 0 (arbitrary units), positive ion voltage 3500 V and negative ion voltage 2500 V. Nitrogen was used as the sheath gas and auxiliary gas. The LC-MS/MS data were

acquired in the selected reaction-monitoring (SRM) mode. The resolutions of Q1 and Q3 were set as 0.7 FWHM, the CID gas (Argon gas) pressure was 1.5 mTorr. Every analyte has resulted in two SRM transitions, one was selected as the quantifier, and the other as the qualifier. All the transitions are listed in Supplementary Table 2 below. The acquired data were processed using Thermo Xcalibur software (version 4.1.31.9).

Supplementary Table 2: SRM transition parameters for Diels-Alder Product (**3a**)

Analytes	Precursor ion (*m/z*)	Product ion (*m/z*)	Collision energy (V)	Dwell time (ms)	RF Lens (V)	Retention Time (min)
Diels-Alder Product	361.15	152.15	14	198.717	51	4.55
		149.05	24	198.717		
Tri-ethyl phosphate (IS)	183.15	126.84	11.9	198.717	35	3.99
		98.98	19.4	198.717		

b) Stereoselectivity study:

Chromatographic separations of Diels-Alder reaction product were achieved on a Surveyor UHPLC system (Thermo Scientific, Germany) consisting of a quaternary gradient pump, an auto-injector and an in-line degasser. The column compartment was maintained at a temperature of 25 °C. The chromatographic separation method was developed in normal phase mode with chiral column (0.46 × 25 cm; Chiralpak AD-H, Daicel Chemical Ind., Ltd.). Varying the selectivity determination factors such as pH of the mobile phase, ratios of polarity change and flow rate optimized the method. Finally, the acceptable separation was achieved using 0.1% TFA in isopropanol (A) and 0.1% TFA in hexane (B) in the ratio of 30:70, v/v with a flow rate of 0.6 mL min^{-1} in isocratic elution mode. The column was equilibrated with 20 column volumes of mobile phase at the composition prior to sample injection and the injection volume was 1µl.

Mass spectral analysis was carried out on Thermo Orbitrap Exactive mass spectrometer (Thermo Scientific, Bremen, Germany). The eluent from the UHPLC system was directed into the mass spectrometer via heated electrospray ionization (HESI) interface and operated in the positive ion mode. The mass spectrometer was calibrated before analysis using the manufacturer's calibration solution (ProteoMass LTQ/FN-Hybrid ESI Pos. Mode Cal Mix, SUPELCO) to obtain the mass range in external calibration mode. Parameters of the ion source were as follows: ion spray voltage 4.20 kV, heater temperature 275 °C, capillary temperature 320 °C, capillary voltage 67.50 V, tube lens voltage 140 V, skimmer voltage 20 V, sheath gas flow 45 (arbitrary units), auxiliary gas flow 10 (arbitrary units) and sweep gas 0 (arbitrary units). Nitrogen was used as the sheath and auxiliary

gas in the ion source. The instrument was operated in full scan FTMS over the range *m/z* 350-400 at a resolving power of 1,00,000 (full width at half maximum). Mass spectral data were processed using Thermo Xcalibur software (version 2.2).

Supplementary Figure 4: APCI-MS (negative mode) spectrum of **1a** ([M-H]$^-$; *m/z* 260.42) and **1b** ([M-H]$^-$; *m/z* 246.37) . ESI-HRMS spectra of **2** ([M+H]$^+$; *m/z* 100.0756, [M+Na]$^+$; *m/z* 411.1889), **3a** ([M+H]$^+$; *m/z* 361.1769.0756, [M+Na]$^+$; *m/z* 383.1580), **3b** ([M+H]$^+$; *m/z* 347.1593, [M+Na]$^+$; *m/z* 369.1410), **4** ([M+H]$^+$; *m/z* 128.1064, [M+Na]$^+$; *m/z* 150.0883), **5** ([M+H]$^+$; *m/z* 142.1221, [M+Na]$^+$; *m/z* 164.0676), **6** ([M+H]$^+$; *m/z* 389.2070, [M+Na]$^+$; *m/z* 411.1889), **7** ([M+H]$^+$; *m/z* 403.2216, [M+Na]$^+$; *m/z* 425.1670).

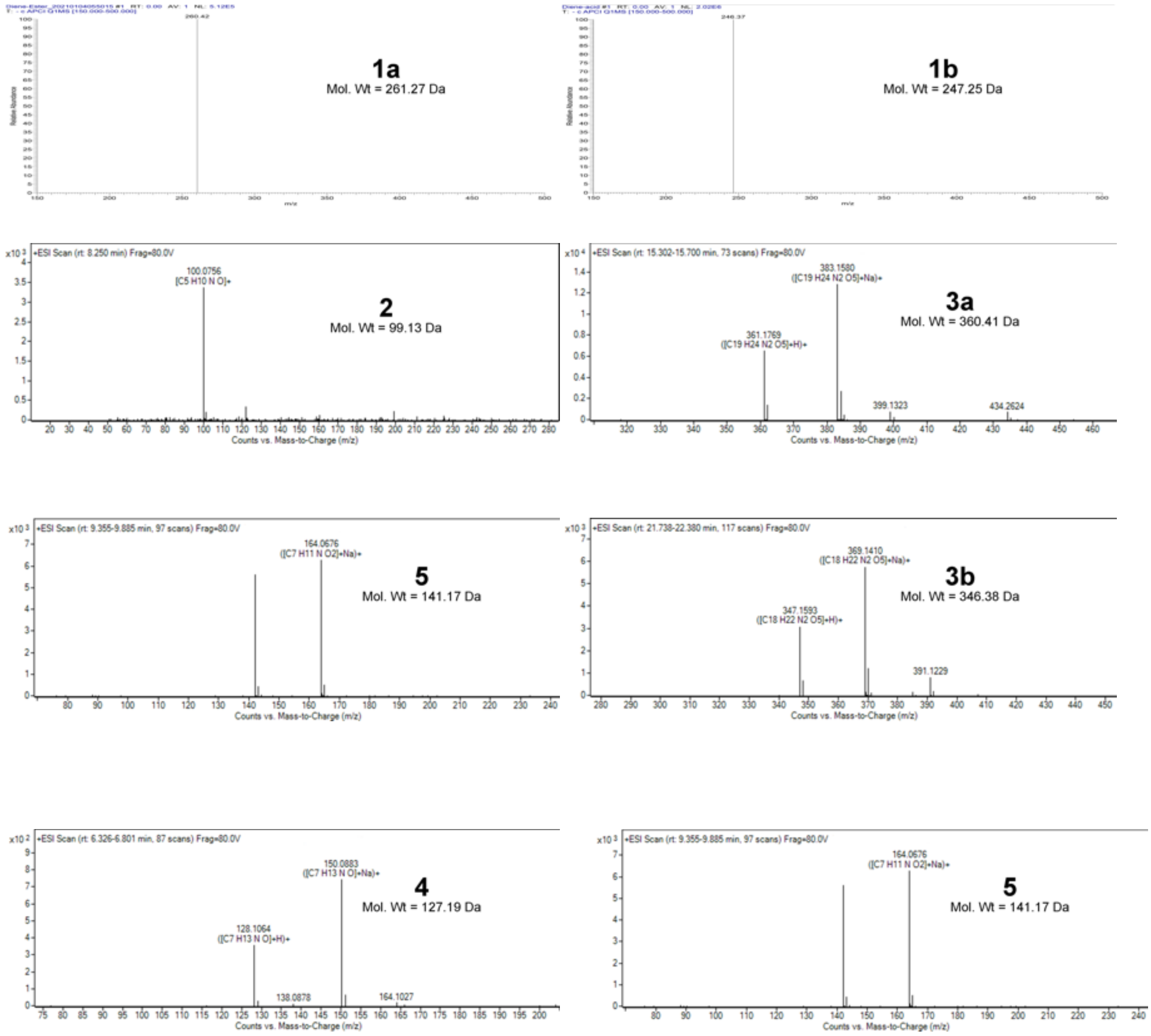

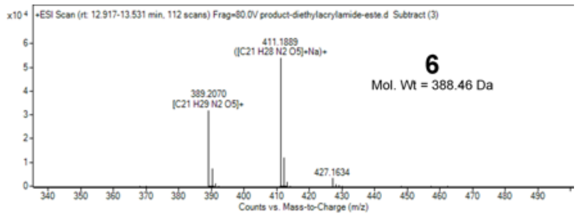

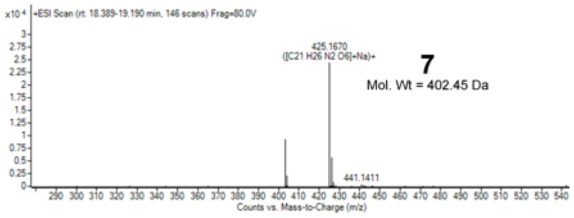

7. Diels-Alder reaction by DAses

The Diels-Alder reaction was performed in PBS buffer with 1 mM of Diene (**1a**), 10 mM of dienophile (**2**), 4% DMSO (vol/vol) and 300 µM of an enzyme i.e. AbnU or PyrI4 with 10% glycerol (volume 100 µl). In the same way, control reaction (background) was carried out in the absence of the enzyme. Reactions were incubated at 37 °C for 2.5 h and quenched with 250 µl ice-cold acetonitrile and then kept at -80 °C for 10 minutes. Acetonitrile layer was separated (200 µl) and internal standard (triethyl phosphate, 275 nM) was added prior to Liquid Chromatography-Selected Reaction Monitoring (LC-SRM) analysis. Standard DA product was also spiked in the buffer without an enzyme and followed the above procedure. Calculated recovery percentage was 97 %. For the absolute quantification, the standard plot was drawn ($R^2 = 0.99$) with the authentic product concentrations ranging from 5.6 nM to 2.22 µM.

8. Kinetic Analysis

Diels alder reactions were performed in PBS buffer at 37°C with 4% DMSO (vol/vol) and 40µM of PyrI4/20 µM of AbnU with 10% glycerol. Diene concentrations were fixed (50, 100, 200, 450 and 600 µM for AbnU and 150, 200, 300, 400 and 600 µM for PyrI4) with varying concentration of dienophile (0, 6, 12, 20, 60, and 100 mM for AbnU and 5, 10, 20, 40, 100 and 150 mM for PyrI4) in the presence or absence of enzyme. Reaction was then incubated for 2.5 h before quenching the reaction with ice-cold acetonitrile and LC-SRM measurement as described above. The experimental data was analysed as described in the previous report [6].

9. Stereoselectivity of Diels-Alderases

Diels-Alder reactions were performed in PBS buffer at 37 °C with 4% DMSO (vol/vol) and 300 µM of enzymes (AbnU, PyrI4, PyrI4-Δ10, and mutant proteins) with 10% glycerol. A reaction with 1 mM of diene and 10 mM dienophile was run for 165 h with and without enzyme.[7, 8] Both the reactions were quenched with an equal volume of a solution of acetonitrile:water (80:20) with 0.1% formic acid[6]. The above mixture was incubated for 5 minutes and half the volume of ethyl acetate was added for the extraction of product (organic layer formed by centrifuge). Extracted product in

ethyl acetate was dried under a stream of nitrogen gas and then analyzed by chiral LC-HRMS as described above.

Supplementary Figure 5: Chiral HPLC spectra of synthetic products. Stereoselectivity pattern for all the ortho isomers (*exo*-3S,4S, *exo*-3R,4R, *endo*-3S,4R and *endo*-3R,4S) of **3a** and **3b** separated by Chiral-HPLC as reported in the literature. Synthesis of **3a** and **3b** used in this experiment is described later in this document. Retention time and the pattern were used as a standard for all the stereoselectivity experiments. We have used the previous literature in assigning the individual peaks to a specific isomer.[7]

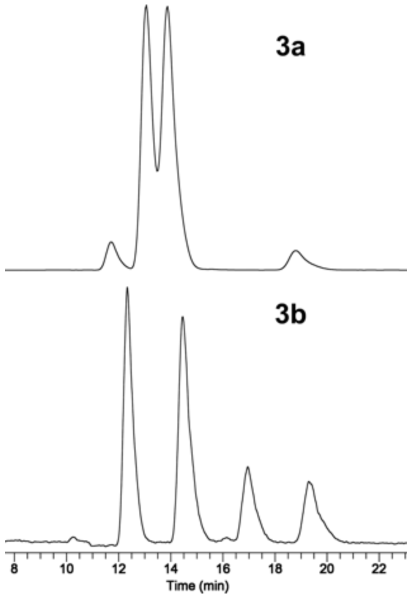

Supplementary Table 3: Integrated peak areas for the stereoselectivity data a) AbnU with **3a,** b) AbnU with **3b** c) PyrI4 with **3a** d) PyrI4-Δ10 with **3a** e) PyrI4 with **3b.**

a

Integrated Area of Peaks				
	Exo (3S,4S)	*Exo* (3R,4R)	*Endo* (3S,4R)	*Endo* (3R,4S)
**3a** (synthetic product)	5901803	35234229	37623516	7500314
Background reaction	538514	736272	625306	731288
AbnU + **1a** + **2**	3012412	186047	308059	365835
AbnU Enhancement	2473898	-550225	-317247	-365453

Relative Peak Area				
	Exo (3S,4S)	*Exo* (3R,4R)	*Endo* (3S,4R)	*Endo* (3R,4S)
**3a** (synthetic product)	6.84%	40.84%	43.62%	8.70%
Background reaction	20.47%	27.98%	23.76%	27.79%
AbnU + **1a** + **2**	77.79%	4.80%	7.96%	9.45%
AbnU Enhancement	199.35%	-44.34%	-25.56%	-29.45%

b

Integrated Area of Peaks				
	Exo (3S,4S)	*Exo* (3R,4R)	*Endo* (3S,4R)	*Endo* (3R,4S)
**3b** (synthetic product)	11988195	12913001	3763906	3497066
Background reaction	1613043	494691	1521197	1588433
AbnU + **1b** + **2**	8824441	1545087	4286487	4102267
AbnU Enhancement	7211398	1050396	2765290	2513834

Relative Peak Area				
	Exo (3S,4S)	*Exo* (3R,4R)	*Endo* (3S,4R)	*Endo* (3R,4S)
**3b** (synthetic product)	37.27%	40.15%	11.70%	10.87%
Background reaction	30.92%	9.48%	29.16%	30.45%
AbnU + **1b** + **2**	47.04%	8.25%	22.85%	21.87%
AbnU Enhancement	53.26%	7.76%	20.42%	18.56%

c

Integrated Area of Peaks				
	Exo (3S,4S)	*Exo* (3R,4R)	*Endo* (3S,4R)	*Endo* (3R,4S)
**3a** (synthetic product)	5901803	35234229	37623516	7500314
Background reaction	65727261	22433920	34291872	96049331
PyrI4 + **1a** + **2**	164308250	37443568	52740078	67583575
PyrI4 Enhancement	98580989	15009648	18448206	-28465756

Relative Peak Area				
	Exo (3S,4S)	*Exo* (3R,4R)	*Endo* (3S,4R)	*Endo* (3R,4S)
**3a** (synthetic product)	6.84%	40.84%	43.62%	8.70%
Background reaction	30.08%	10.27%	15.69%	43.96%
PyrI4 + **1a** + **2**	51.01%	11.63%	16.38%	20.98%
PyrI4 Enhancement	95.18%	14.49%	17.81%	-27.48%

d

Integrated Area of Peaks				
	Exo (3S,4S)	*Exo* (3R,4R)	*Endo* (3S,4R)	*Endo* (3R,4S)
**3a** (synthetic product)	5901803	35234229	37623516	7500314
Background reaction	65727261	22433920	34291872	96049331
PyrI4-Δ10 + **1a** + **2**	126446477	33146744	45113286	56019695
PyrI4-Δ10 Enhancement	60719216	10712824	10821414	-40029636

Relative Peak Area				
	Exo (3S,4S)	*Exo* (3R,4R)	*Endo* (3S,4R)	*Endo* (3R,4S)
**3a** (synthetic product)	6.84%	40.84%	43.62%	8.70%
Background reaction	30.08%	10.27%	15.69%	43.96%
PyrI4-Δ10 + **1a** + **2**	48.50%	12.71%	17.30%	21.49%
PyrI4-Δ10 Enhancement	143.80%	25.37%	25.63%	-94.80%

e

Integrated Area of Peaks				
	Exo (3S,4S)	*Exo* (3R,4R)	*Endo* (3S,4R)	*Endo* (3R,4S)
**3b** (synthetic product)	11988195	12913001	3763906	3497066
Background reaction	1613043	494691	1521197	1588433
PyrI4 + **1b** + **2**	4115294	963833	2769367	3229729
PyrI4 Enhancement	2502251	469142	1248170	1641296

Relative Peak Area				
	Exo (3S,4S)	*Exo* (3R,4R)	*Endo* (3S,4R)	*Endo* (3R,4S)
**3b** (synthetic product)	37.27%	40.15%	11.70%	10.87%
Background reaction	30.92%	9.48%	29.16%	30.45%
PyrI4 + **1b** + **2**	37.15%	8.70%	25.01%	29.15%
PyrI4 Enhancement	42.69%	8.00%	21.30%	28.00%

Supplementary Figure 6: Stereoselectivity data for background (non-enzymatic), AbnU, PyrI4 and PyrI4-Δ10 (only for **3a**) represented by overlay of the extracted ion chromatogram (EIC) of a) **3a** [*m/z* 361 [M+H]$^+$ and *m/z* 383 [M+Na]$^+$] and b) **3b** [*m/z* 347 [M+H]$^+$ and *m/z* 369 [M+Na]$^+$] respectively.

a)

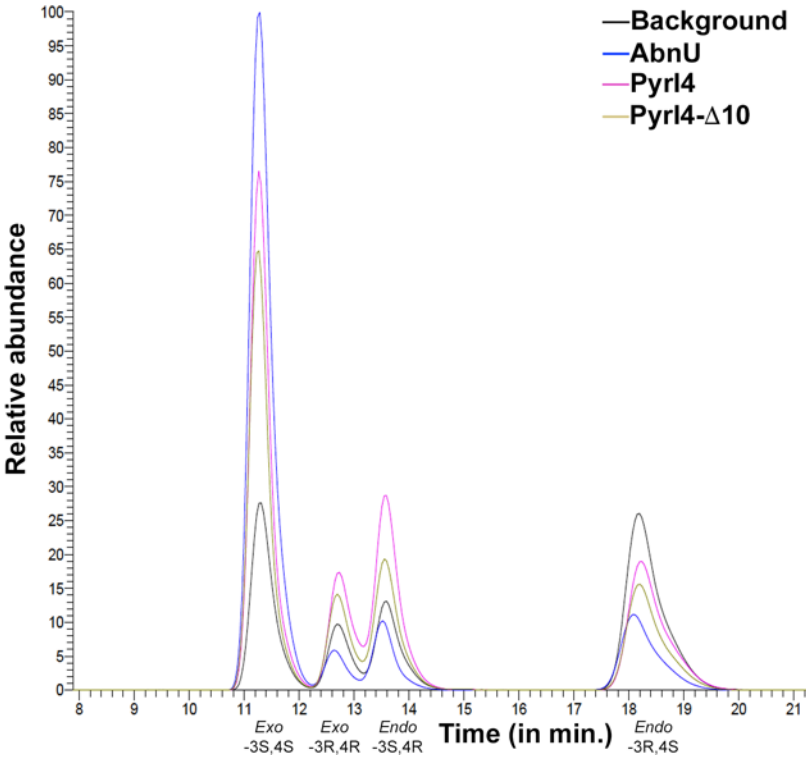

b)

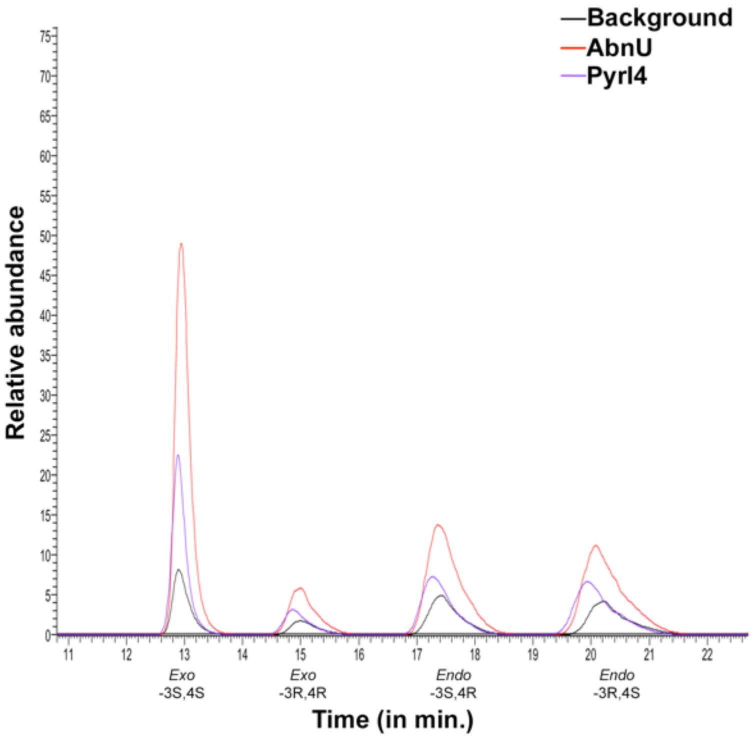

10. Diffraction data collection and structure determination

Apo PyrI4 crystals for diffraction data collection were mounted in an appropriately sized litholoops (Molecular Dimensions Ltd) and directly flash-cooled in liquid nitrogen prior to analysis. PyrI4-Δ10 protein crystals were submerged in mother liquor supplemented with 10% glycerol followed by flash freezing in liquid nitrogen prior to analysis. The diffraction data for the PyrI4 and PyrI4-Δ10-**3a** complex were collected at the home source on a MARS CCD detector using a home source X-ray diffractometer (Rigaku Micromax-007) with R-axis IV++ detector. AbnU protein crystal was submerged in mother liquor supplemented with 15% ethylene glycol followed by flash freezing in liquid nitrogen prior to analysis. X-ray data for AbnU were collected on beamline XRD2 at Elettra synchrotron, Trieste, Italy.

Diffraction data were processed using XDS.[9] Data were further scaled and merged using AIMLESS as implemented within the CCP4 suite of programs.[10, 11] About 5% of the data were set aside for the calculation of R_{free}. Molecular replacement was performed by MOLREP using PDB ID: 5BTU for PyrI4 as a model while PDB ID: 5DYV for AbnU as a model in the CCP4 suite of programs.[12] Further several iterative rounds of manual model building and refinement were carried out using COOT and REFMAC5.[13, 14] Crystallographic data for AbnU and PyrI4-Δ10 are summarized in the table below (Supplementary Table 4). Unfortunately, PyrI4 crystals did not diffract better than 3.0 Å and had very high mosaicity which did not permit us to solve the structure.

Supplementary Table 4: Crystallographic table of AbnU and PyrI4-Δ10

	**AbnU**	**PyrI4-Δ10-3a**
Wavelength	0.974	1.54
Resolution range	41.34 - 2.003 (2.074 - 2.003)	46.04 - 2.601 (2.694 - 2.601)
Space group	P 6(4)22	P 4(3)2(1)2
Unit cell	104.44 104.44 67.66 90 90 120	102.96 102.96 168.80 90 90 90
Total reflections	552015 (51514)	290143 (28180)
Unique reflections	15136 (1464)	28575 (2764)
Multiplicity	36.5 (35.2)	10.2 (10.2)
Completeness (%)	99.89 (99.19)	99.68 (98.36)
Mean I/sigma(I)	42.85 (7.94)	21.18 (5.76)
Wilson B-factor	34.32	40.67
R-merge	0.06271 (0.5703)	0.0861 (0.3639)
R-meas	0.06361 (0.5787)	0.09072 (0.3831)
R-pim	0.01055 (0.09681)	0.0281 (0.1181)
CC1/2	1 (0.982)	0.999 (0.949)
CC*	1 (0.995)	1 (0.987)
Reflections used in refinement	15134 (1464)	28560 (2764)
Reflections used for R-free	724 (76)	1402 (120)
R-work	0.19	0.21
R-free	0.24	0.27
Number of non-hydrogen atoms	1265	5053
macromolecules	1099	4869
ligands	96	134
solvent	70	50
Protein residues	134	652
RMS(bonds)	0.04	0.29
RMS(angles)	2.03	5.08
Ramachandran favored (%)	98.48	96.12
Ramachandran allowed (%)	1.52	3.88
Ramachandran outliers (%)	0.00	0.00
Rotamer outliers (%)	5.22	10.44
Clashscore	5.02	9.29
Average B-factor	39.30	44.08
macromolecules	37.48	43.33
ligands	55.20	76.45
solvent	45.99	30.60
PDB ID	7DVI	7DVK

Supplementary Figure 7: Energy minimized three-dimensional structure and molecular surface representation (dot) of **3a** a) linear *Exo*-3S,4S and b) U-shaped *Endo*-3R,4S generated by ChemDraw version 19.

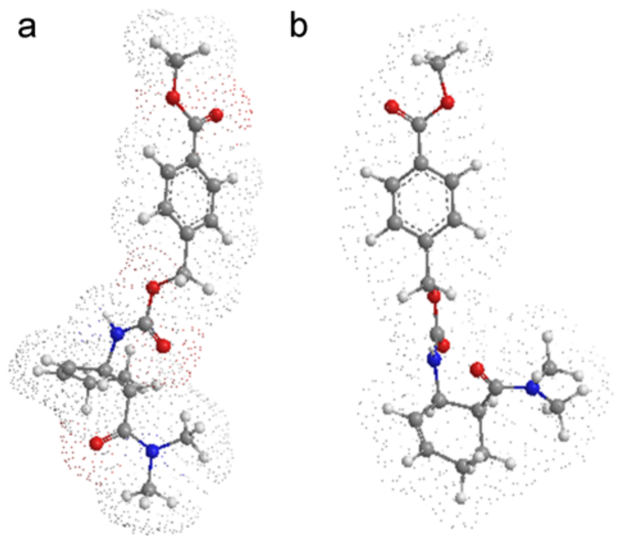

Exo (3S,4S) (**3a**)   *Endo* (3R,4S) (**3a**)

11. Confirmation of product bound PyrI4-Δ10 via mass spec

Crystals of PyrI4-Δ10 co-crystallized with **3a** were examined for the presence of the product. Crystals were thoroughly washed in the well solution to remove any ligand bound on the surface. Then the crystals were crushed in ethyl acetate and mixed thoroughly to separate the aqueous layer with protein and buffer, and organic layer. The organic layer was subjected to mass spectral analysis (Supplementary Figure 8a and 8b).

Supplementary Figure 8: Mass spectral analysis of product **3a** extracted from PyrI4-Δ10-**3a** co-crystal a) ESI-HRMS spectrum data depicting the presence of product **3a** in PyrI4-Δ10-**3a** co-crystal b) Chiral Liquid Chromatography-Selected Reaction Monitoring (LC-SRM) analysis of product **3a** in PyrI4-Δ10-**3a** co-crystal showing the presence of both the exo adduct with the retention time matching with the synthetic standard **3a**.

a

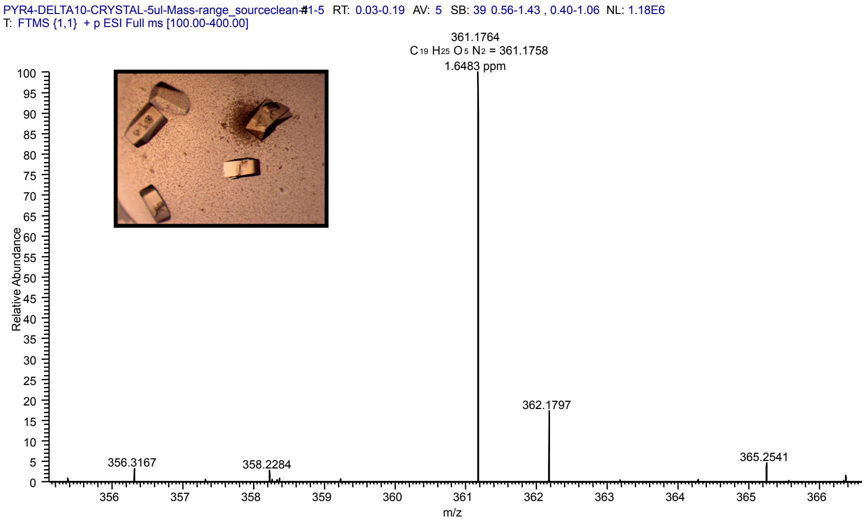

b

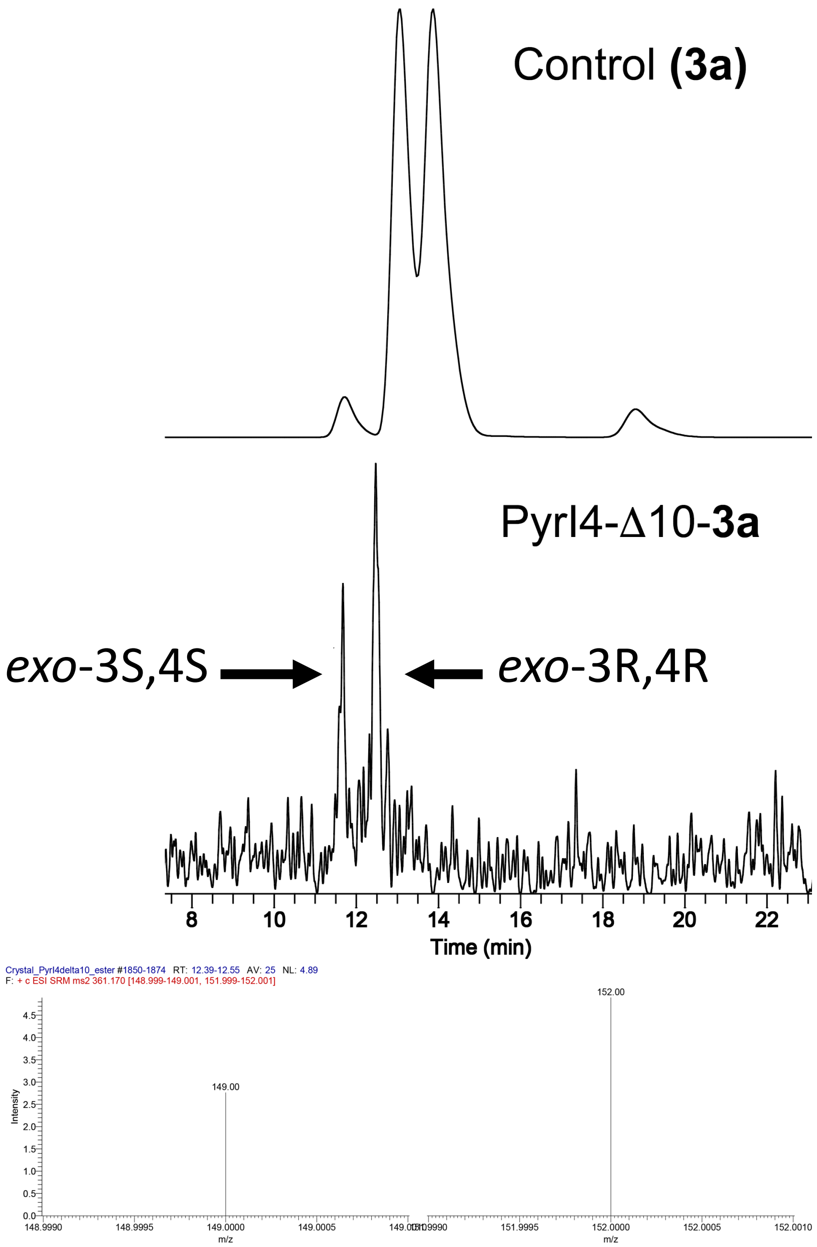
Control (**3a**)
PyrI4-Δ10-**3a**
exo-3S,4S
exo-3R,4R
8
10
12
14
16
18
20
22
Time (min)
Crystal_PyrI4delta10_ester #1850-1874 RT: 12.39-12.55 AV: 25 NL: 4.89
F: + c ESI SRM ms2 361.170 [148.999-149.001, 151.999-152.001]
149.00
152.00
Intensity
m/z

Supplementary Figure 9: Crystal structure of AbnU determined at 2.0 Å resolution depicted by ribbon and the surface representation of dimer of AbnU.

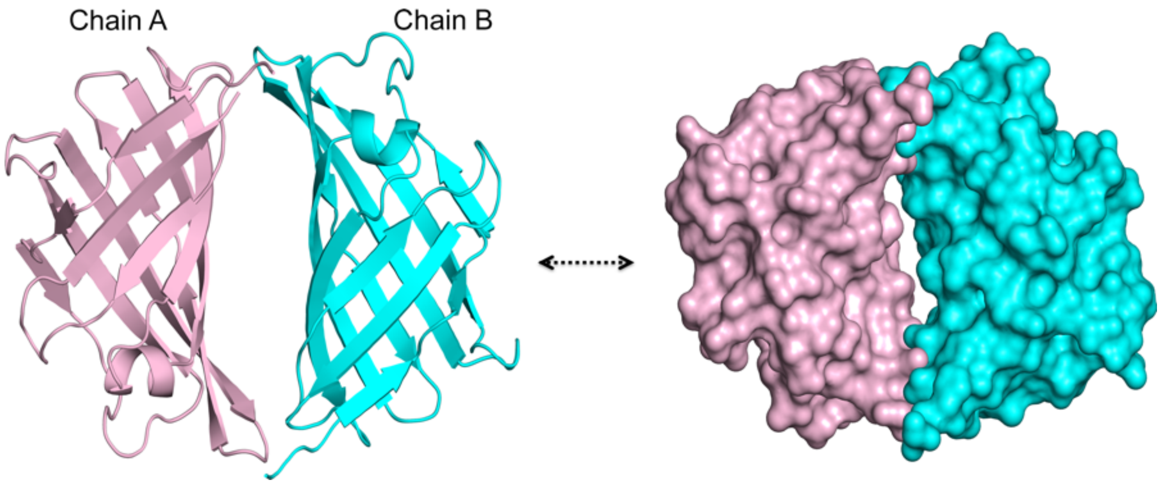

12. Tryptophan fluorescence spectroscopy:

All tryptophan fluorescence spectroscopy experiments were performed using Varioskan FLASH multimode reader (Thermo Scientific) and 96-well black flat bottom microtiter plate (Corning Inc., USA) at ambient temperature. The excitation wavelength was fixed to 285 nm and emission spectra were collected between 300 and 500 nm with a slit width of 5 nm.

To study the AbnU-ligand interactions, recombinant AbnU protein at 20 μM was equilibrated in PBS buffer prior to experiment. Ligands included the Dienes **1a** and **1b**, Dienophile **2** and the Diels-Alder adduct **3a**. AbnU-ligand interaction was tested with increasing concentrations of **1a** and **1b** from 0 μM to 3000 μM, **2** from 0 mM to 1000 mM and Diels-Alder product **3a** from 0 μM to 3000 μM. Control buffer titration experiments were always performed in parallel to ensure background subtraction, if any.

The cumulative effect of the binding of both the substrates was analyzed by titrating fixed concentration of **1a** at 500 μM and **2** at 5 mM in the presence or absence of 20 μM AbnU. Control fluorescence of the individual substrate and the buffer titration was performed in triplicate.

Triplicate experimental data were analyzed for the background subtraction and was further analyzed by using nonlinear regression with 'One Site - Specific Binding' model with the following equation:

$$Y = B_{max} * X/(K_D + X)$$

where X is the ligand concentration, Y is the fluorescence intensity, B_{max} is the maximum specific binding and K_D is the equilibrium binding constant. K_D values were determined by fitting the data to the above equation in GraphPad Prism 8.1.0.

Supplementary Figure 10: Intrinsic tryptophan fluorescence (λ_{ex}=285 nm, λ_{em}=330 nm) profile for a) AbnU in the presence and absence of DMSO showing no effect on its fluorescence b) Comparison of tryptophan fluorescence between AbnU and PyrI4

a b

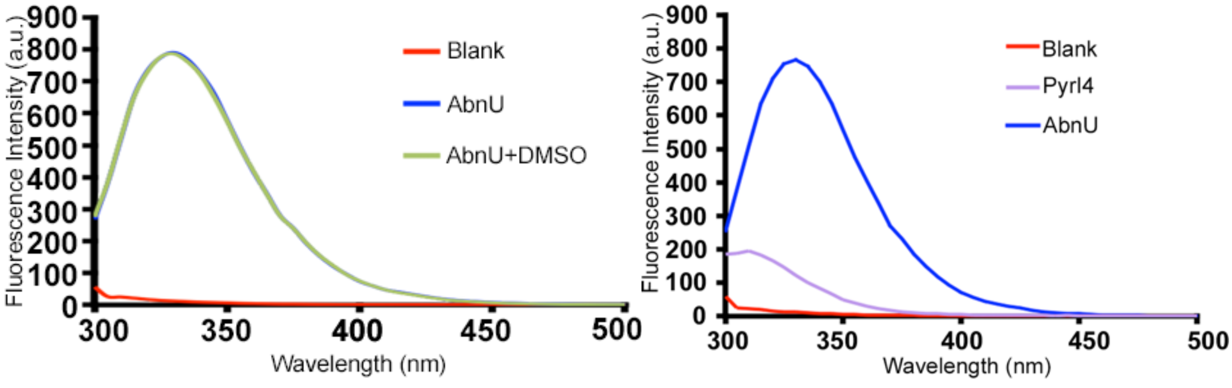

13. Substrate Scope

Diels alder reactions were performed in PBS buffer at 37°C with 4% DMSO (vol/vol), fixed concentration of **1a** (0.2 mM) and different dienophiles namely **2**, **4** and **5** (each 10 mM) were incubated with and without 60 uM of AbnU and PyrI4 in 10% glycerol. The reaction was incubated for 2.5 hr and quenched with ice-cold acetonitrile. Relative quantification of the analytes was carried out by using UHPLC coupled with ESI-SRM with the flow rate as 0.4 ml/min. The elution gradient was modified and optimized at: (time in min/proportion of solvent B): 0-0.3/5, 0.3-4/95, 4-5.9/95, 5.9-6.0/5, and 6.0-8.0/5. Same SRM parameters (Supplementary Table 4) were used for the analyte **(3a)** without the internal standard. Data were analyzed by Thermo Xcalibur software.

Supplementary Figure 11: Substrate selectivity of AbnU and PyrI4 for different dienophiles namely **2, 4 and 5.** Data shown here represent the comparison between background (non-enzymatic) and enzyme catalyzed activity. All error bars reflect the standard deviation obtained from independent triplicate experiments.

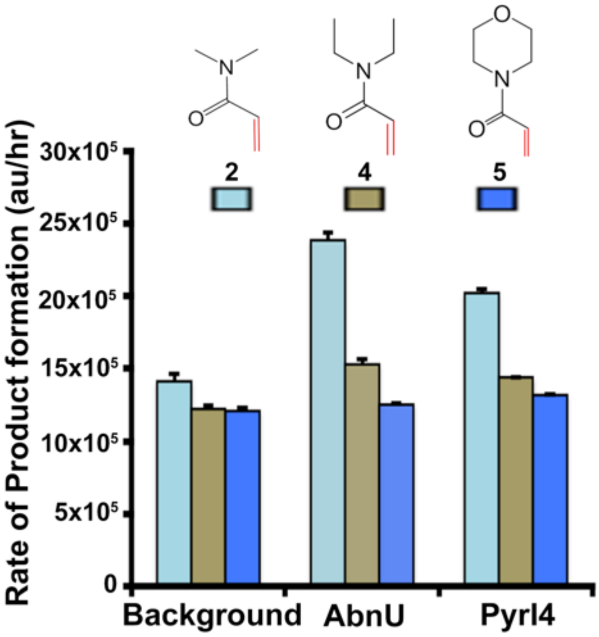

14. Chemical synthesis dienes, dienophiles and products

General Procedure:

Methyl (*E*)-4-(buta-1, 3-dien-1-ylcarbamoyl)oxy)methyl)benzoate (1a): All the synthetic protocols were based on the previous report.[7] 2,4-pentadienoic acid (**A**) (1 eq) dissolved in 30 mL of toluene and *N,N*-diisopropylethylamine was added drop wise at room temperature under nitrogen. The resulting clear solution was stirred and cooled to 0 °C in ice-bath. A solution of ethyl chloroformate (2 eq) in 90 mL of toluene was added over 30 minutes while the temperature was maintained at 0 °C. Stirring was continued for 30 minutes at 0 °C, after sodium azide (2 eq) was added. The solution was stirred for additional 30 minutes at 0 °C and poured into separatory funnel containing 150 mL of ice-water. The acylazide was isolated in mother liquor and dried over Na_2SO_4.

Above isolated acylazide (toluene) treated with methyl 4-(hydroxymethyl)benzoate (**B**) (1.2 eq) stirred at 110 °C. The reaction was monitored by TLC and after the indicated reaction time; first the cooled mixture and quenched with saturated ammonium chloride solution and partitioned between ice-cold water (30 mL) and ethyl acetate (50 mL). The organic layer was separated, and the aqueous layer was extracted with ethyl acetate (2 × 30 mL). The combined organic layer was washed with brine and dried over Na_2SO_4. The solvent was removed in *vacuo*. The residue was purified by column chromatography on silica gel to afford the desired product (**1a**) (Supplementary Scheme 1).

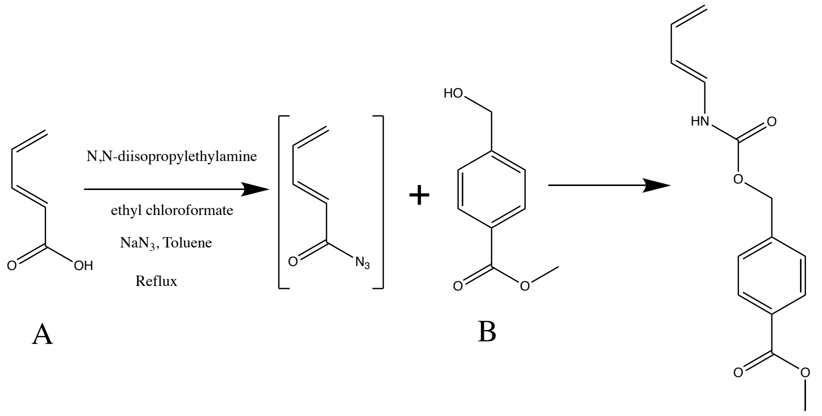

1a

Supplementary Scheme 1. Synthesis of **1a**

Diels Alder reaction general procedure.

Methyl (*E*)-4-(buta-1, 3-dien-1-ylcarbamoyl)oxy)methyl)benzoate (**1a**) (1eq), dissolved in 10 mL toluene and dienophile (**2**) (1.2 eq) were added at room temperature and the reaction was stirred under reflux conditions for 4 days. The reaction was monitored by TLC and after the completion of the reaction indicated; first the mixture was cooled and quenched with saturated ammonium chloride solution and partitioned between ice-cold water (30 mL) and ethyl acetate (50 mL). The organic layer was separated, and the aqueous layer was extracted with ethyl acetate (2 × 30 mL). The combined organic layer was washed with brine and dried over Na_2SO_4. The solvent was removed in *vacuo*. The residue was purified by column chromatography on silica gel to afford the desired product (**3a**) (Supplementary Scheme 2).

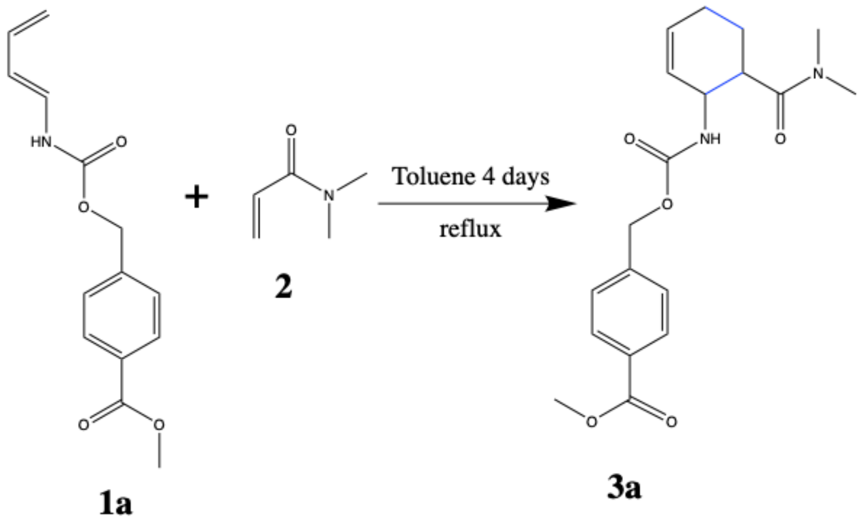

Supplementary Scheme 2. Synthesis of **3a**

Ester hydrolysis

A solution of $LiOH.H_2O$ (3 eq) in 5 mL of water was added to a solution of **1a** or **3a** in 15 mL of THF. The biphasic system was stirred at room temperature for 24 h until it became homogeneous. The solution was acidified by the addition of 1M HCl, and extracted with ethyl acetate (2 x 30 mL). The organic layer dried over Na_2SO_4 and evaporated in *vacuo*. The yellow crude product was purified by flash chromatography on silica gel to give as a white solid (**1b** or **3b**) (Supplementary Scheme 3).

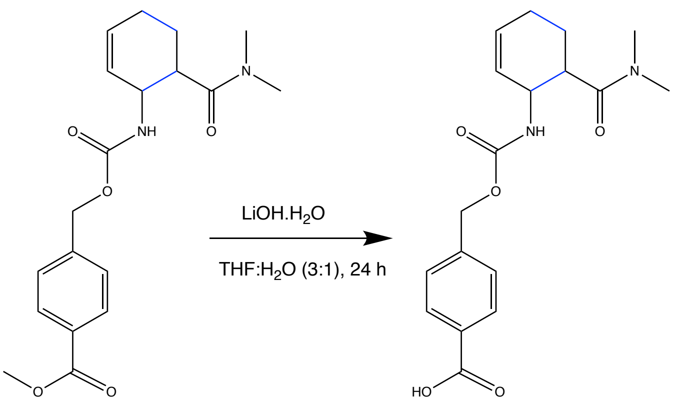

3a **3b**

Supplementary Scheme 3. Synthesis of **3b**

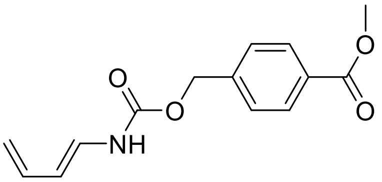

Methyl (*E*)-4-(buta-1, 3-dien-1-ylcarbamoyl)oxy)methyl)benzoate (1a) White solid, IR (KBr): 3281, 3016, 2958, 2930, 1726 (C=O), 1697 (C=O) cm^{-1}; ^{1}H NMR (400 MHz, $CDCl_3$): **δ** 8.03 (d, *J* = 8.2 Hz, 2H), 7.41 (d, *J* = 8.0 Hz, 2H), 6.79 – 6.70 (m, 1H), 6.54 (d, *J* = 12.3 Hz, 1H), 6.28 (dt, *J* = 17.8, 10.3 Hz, 1H), 5.79 – 5.66 (m, 1H), 5.21 (s, 2H), 5.05 (d, *J* = 19.2 Hz, 1H), 4.93 (d, *J* = 11.0 Hz, 1H), 3.92 (s, 3H); ^{13}C NMR (100 MHz, $CDCl_3$) δ 166.74, 153.12, 140.85, 134.31, 129.91, 127.70, 126.77, 113.81, 112.55, 66.56, 52.23; LC-MS: $(M+H)^+$ = 262.10 (Supplementary Figure 12a and 12b).

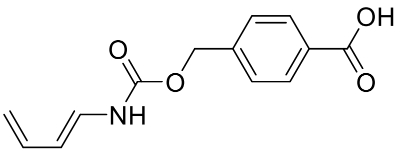

(E)-4-(((Buta-1, 3-dien-1-ylcarbamoyl) oxy) methyl) benzoic acid (1b) White solid, IR (KBr): 3330, 2954, 1702 (C=O), 1659 (C=O) cm^{-1}; ^{1}H NMR (300 MHz, $CDCl_3$+DMSO): **δ** 9.47 (d, *J* = 10.0 Hz, 1H), 8.07 (d, *J* = 8.0 Hz, 2H), 7.49 (d, *J* = 8.0 Hz, 2H), 6.82 – 6.65 (m, 1H), 6.30 (dt, *J* = 16.9, 10.4 Hz, 1H), 5.99 – 5.75 (m, 1H), 5.25 (s, 2H), 5.04 (d, *J* = 16.9 Hz, 1H), 4.89 (d, *J* = 10.3 Hz, 1H), 2.65 (s, 1H); ^{13}C NMR (100 MHz, $CDCl_3$+DMSO) δ 168.53, 153.91, 140.84, 135.23, 131.74, 129.98, 128.35, 127.59, 112.59, 112.15, 66.04. LC-MS: $(M-H)^-$ = 246.10 (Supplementary Figure 13a and 13b).

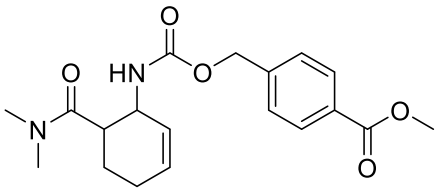

Methyl 4-((((6-(Dimethylamino) cyclohex-2-en-1-yl) carbamoyl) oxy) methyl) benzoate (3a) White solid, IR (KBr): 3287, 3016, 2958, 2930, 1726 (C=O), 1697 (C=O) cm $^{-1}$; ^{1}H NMR (400 MHz, $CDCl_3$): **δ** 8.01 (d, *J* = 8.0 Hz, 2H), 7.39 (d, *J* = 6.1 Hz, 2H), 5.81 (d, *J* = 9.4 Hz, 1H), 5.66 (d, *J* = 8.8 Hz, 1H), 5.11 (dd, *J* = 26.6, 12.7 Hz, 2H), 4.37 (s, 1H), 3.92 (s, 3H), 3.12 (dd, *J* = 25.4, 2.7 Hz, 1H), 2.98 (s, 3H), 2.93 (s, 3H), 2.13 (s, 2H), 1.82 (dd, *J* = 9.4, 5.2 Hz, 2H); ^{13}C NMR (100 MHz, $CDCl_3$) δ 173.77, 166.82, 155.13, 141.87, 129.77, 128.49, 128.34, 127.50, 65.63, 52.18, 50.43, 41.31, 37.22, 35.76, 25.08, 24.24; LC-MS: (M+H)$^{+}$ = 361.15 (Supplementary Figure 14a and 14b).

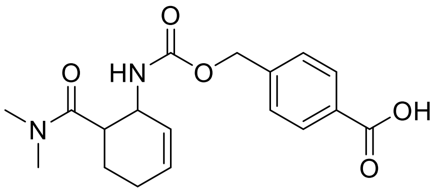

4-((((6-(dimethylamino)cyclohex-2-en-1-yl)carbamoyl) oxy) methyl) benzoic acid (3b) White solid, ^{1}H NMR (300 MHz, $CDCl_3$): **δ** 8.07 (dd, *J* = 16.5, 8.2 Hz, 4H), 7.84 (d, *J* = 7.7 Hz, 1H), 7.52 – 7.37 (m, 4H), 6.11 (t, *J* = 8.8 Hz, 1H), 5.98 – 5.87 (m, 1H), 5.86 – 5.64 (m, 2H), 5.50 – 5.35 (m, 1H), 5.20 – 5.07 (m, 2H), 5.03 (s, 1H), 4.80 (s, 1H), 4.59 – 4.52 (m, 1H), 4.38 – 4.37 (m, 1H), 3.18 (s, 3H), 3.01 (s, 3H), 2.94 (s, 6H), 2.15 (s, 4H), 2.07 – 1.98 (m, 2H), 1.83 (s, 4H), 0.98 – 0.74 (m, 2H); LC-MS: (M+H)$^{+}$ = 347.10 (Supplementary Figure 15).

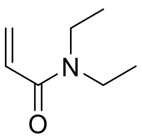

N,N-diethylacrylamide (4) ^{1}H NMR (400 MHz, CDCl3): δ 6.55 (dd, J = 16.7, 10.4 Hz, 2H), 6.34 (dd, J = 16.7, 2.1 Hz, 2H), 5.66 (dd, J = 10.4, 2.1 Hz, 2H), 3.42 (dq, J = 24.5, 7.1 Hz, 9H), 1.18 (dt, J = 15.9, 7.1 Hz, 14H). ^{13}C NMR (101 MHz, CDCl3): δ 165.63, 127.93, 127.53, 42.21, 40.82, 14.85, 13.12. LC-MS: (M+H)+ =128.15 (Supplementary Figure 16a and 16b).

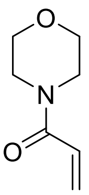

1-morpholinoprop-2-en-1-one (5) ^{1}H NMR (400 MHz, CDCl3): δ 6.62 – 6.50 (m, 1H), 6.31 (d, J = 16.8, 3.8, 1.9 Hz, 1H), 5.72 (d, J = 10.5, 3.5, 1.7 Hz, 1H), 3.64 (d, J = 44.8 Hz, 8H). ^{13}C NMR (101 MHz, CDCl3): δ 165.48, 128.37, 127.02, 77.09, 66.80, 46.22, 42.28. LC-MS: (M+H)+ =142.21 (Supplementary Figure 17a and 17b).

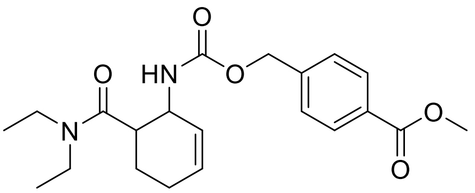

Methyl 4-((((6- (Diethylcarbamoyl) cyclohex-2-en-1-yl) carbamoyl) oxy) methyl) benzoate (6) ^{1}H NMR (400 MHz, $CDCl_3$): **δ** 8.00 (d, *J* = 8.1 Hz, 3H), 7.38 (d, *J* = 7.5 Hz, 3H), 5.81 (d, *J* = 8.0 Hz, 1H), 5.66 (d, *J* = 9.1 Hz, 1H), 5.16 (d, *J* = 12.8 Hz, 3H), 5.04 (d, *J* = 12.9 Hz, 1H), 4.35 (s, 1H), 3.92 (s, 5H), 3.41 (dt, *J* = 14.1, 7.0 Hz, 1H), 3.27 (qd, *J* = 14.0, 7.1 Hz, 4H), 3.10 (s, 1H), 2.13 (t, *J* = 16.7 Hz, 3H), 1.81 (dd, *J* = 8.4, 4.4 Hz, 3H), 1.07 (t, *J* = 7.0 Hz, 8H).^{13}C NMR (101 MHz, $CDCl_3$): **δ** 173.26, 166.83, 155.12, 141.89, 129.85, 129.75, 128.82, 128.13, 127.58, 65.54, 52.18, 50.84, 41.87, 41.37, 40.48, 26.05, 24.34, 14.89, 13.13. LC-MS: $(M+H)^+$ = 389.2071 (Supplementary Figure 18a and 18b).

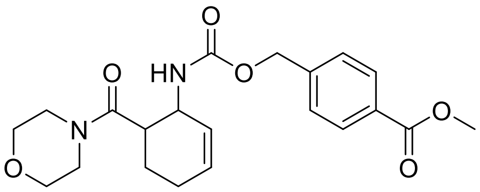

Methyl 4-((((6-(morpholine-4-carbonyl) cyclohex-2-en-1-yl) carbamoyl) oxy) methyl) benzoate (7) ^{1}H NMR (400 MHz, $CDCl_3$): δ 8.01 (d, *J* = 8.2 Hz, 2H), 7.39 (d, *J* = 8.1 Hz, 2H), 5.89 (d, *J* = 9.7 Hz, 1H), 5.72 – 5.65 (m, 1H), 5.27 (d, *J* = 9.7 Hz, 1H), 5.13 (dd, *J* = 27.8, 13.3 Hz, 2H), 4.46 (d, *J* = 4.2 Hz, 1H), 3.91 (s, 3H), 3.80 (dd, *J* = 13.2, 7.1 Hz, 1H), 3.70 – 3.38 (m, 6H), 2.97 (dd, *J* = 12.5, 6.2 Hz, 1H), 2.16 (dd, *J* = 18.1, 4.3 Hz, 1H), 2.08 – 1.99 (m, 1H), 1.87 – 1.78 (m, 2H). ^{13}C NMR (101 MHz, $CDCl_3$): δ 170.94, 166.86, 155.62, 141.92, 130.38, 129.76, 129.60, 127.19, 126.42, 66.62, 66.36, 65.81, 52.18, 46.05, 45.98, 41.99, 40.71, 23.98, 21.45. LC-MS: $(M+H)^+$ = 403.1864 (Supplementary Figure 19a and 19b).

Supplementary Figure 12: a) ^{1}H NMR (400 MHz, $CDCl_3$) Spectrum of compound **1a**

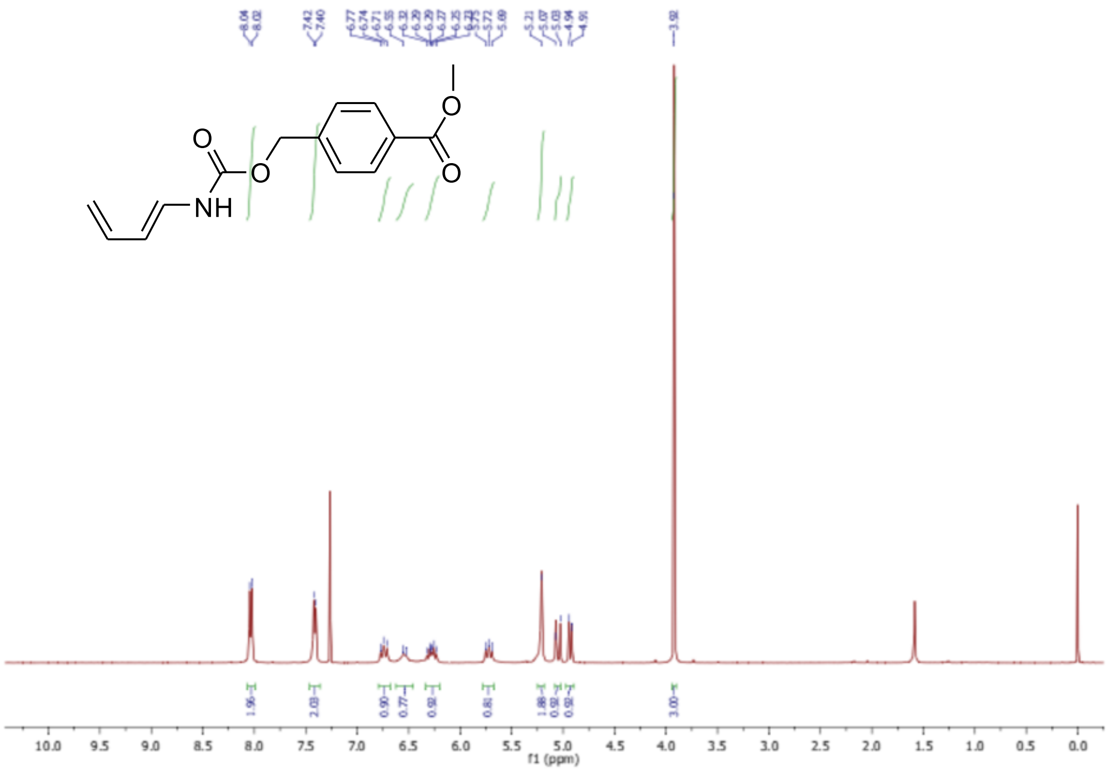

b) ^{13}C NMR (100 MHz, $CDCl_3$) Spectrum of compound **1a**

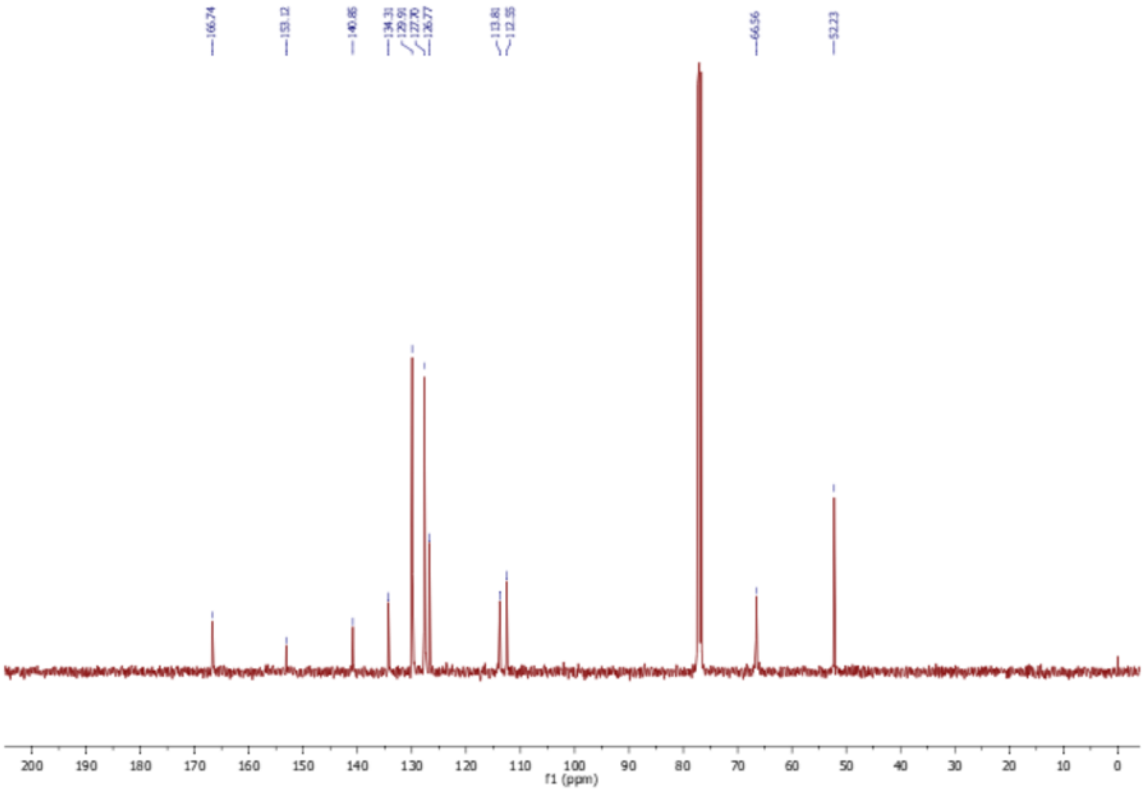

Supplementary Figure 13: a) ^{1}H NMR (300 MHz, $CDCl_3$+DMSO) Spectrum of compound **1b**

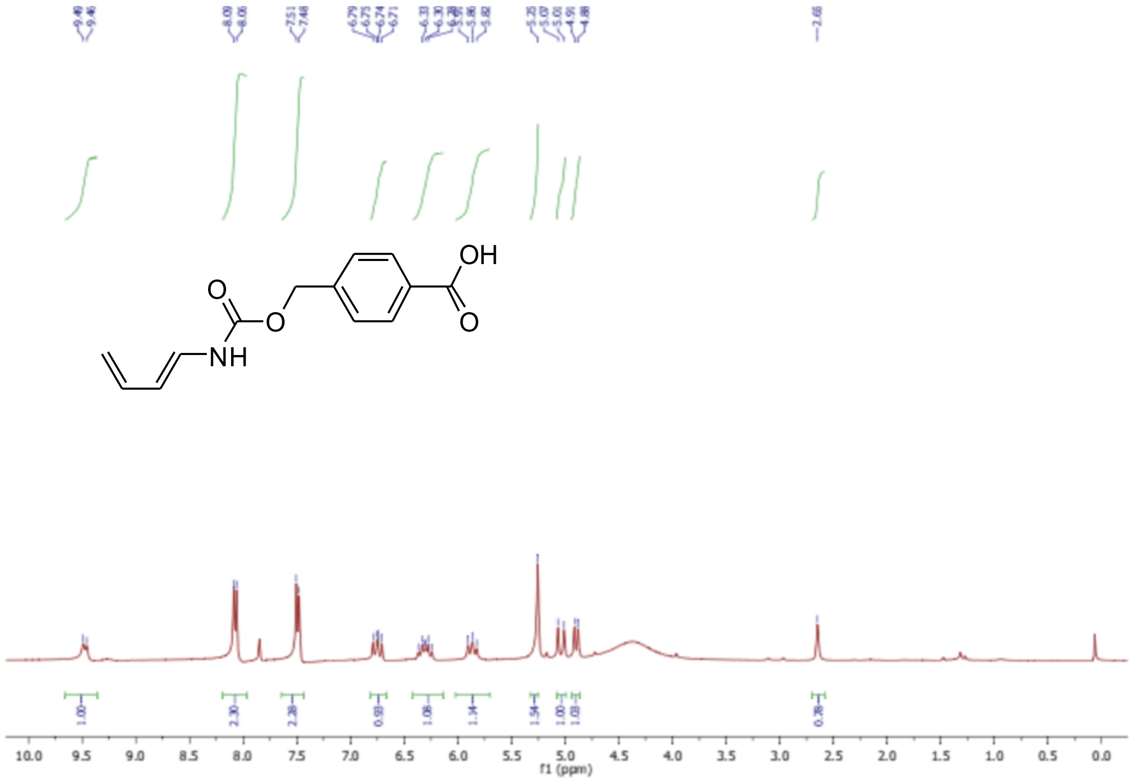

b) ^{13}C NMR (100 MHz, $CDCl_3$+DMSO) Spectrum of compound **1b**

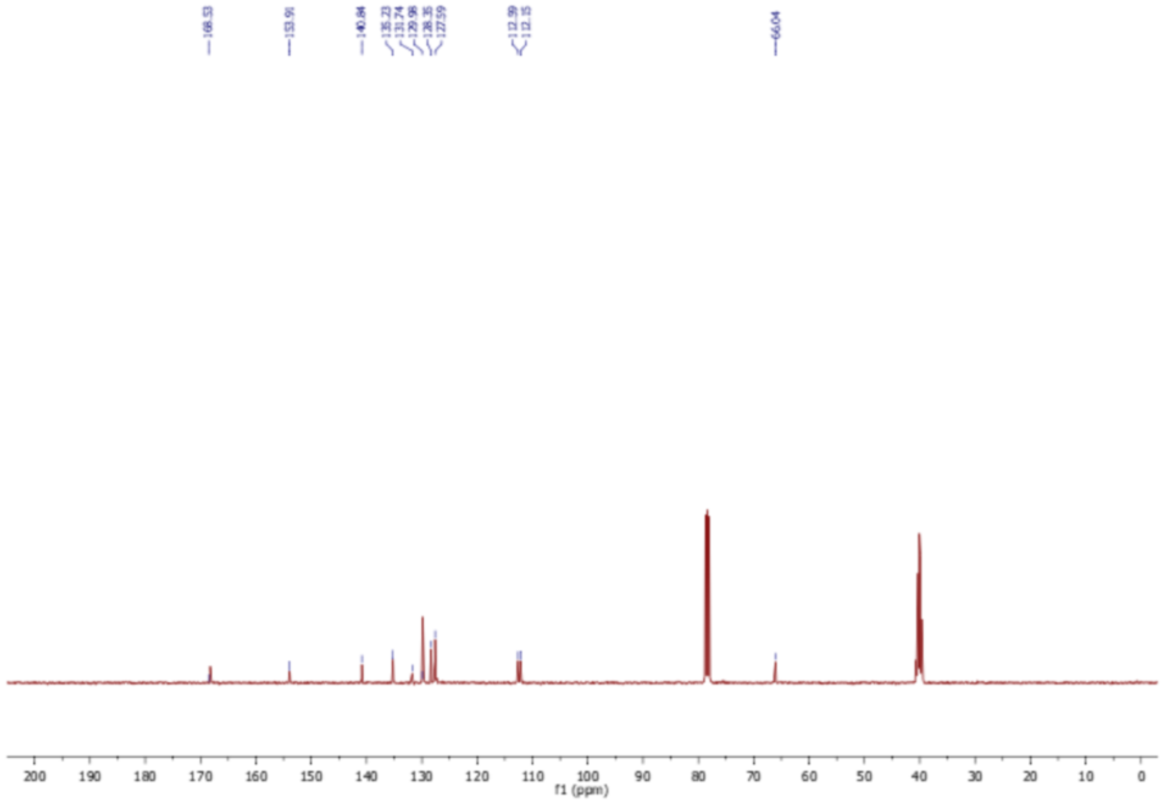

Supplementary Figure 14: a) ^{1}H NMR (400 MHz, $CDCl_3$) Spectrum of compound **3a**

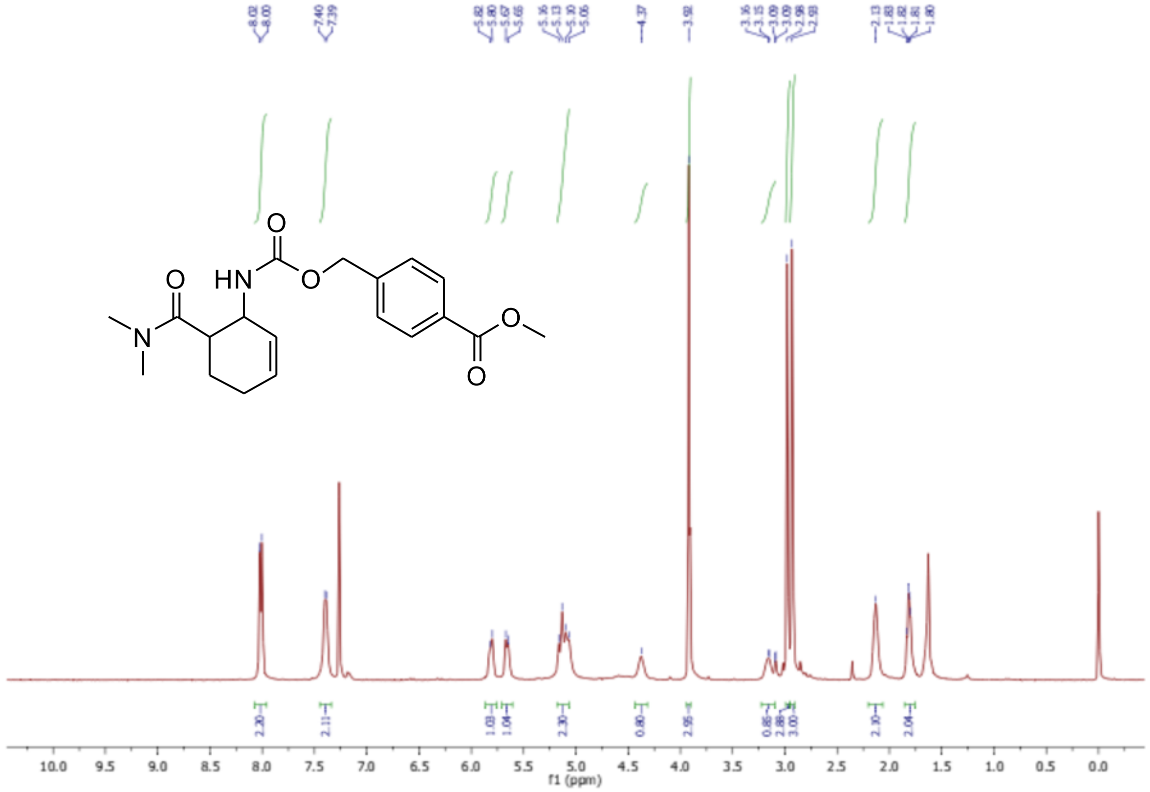

b) ^{13}C NMR (100 MHz, $CDCl_3$) Spectrum of compound **3a**

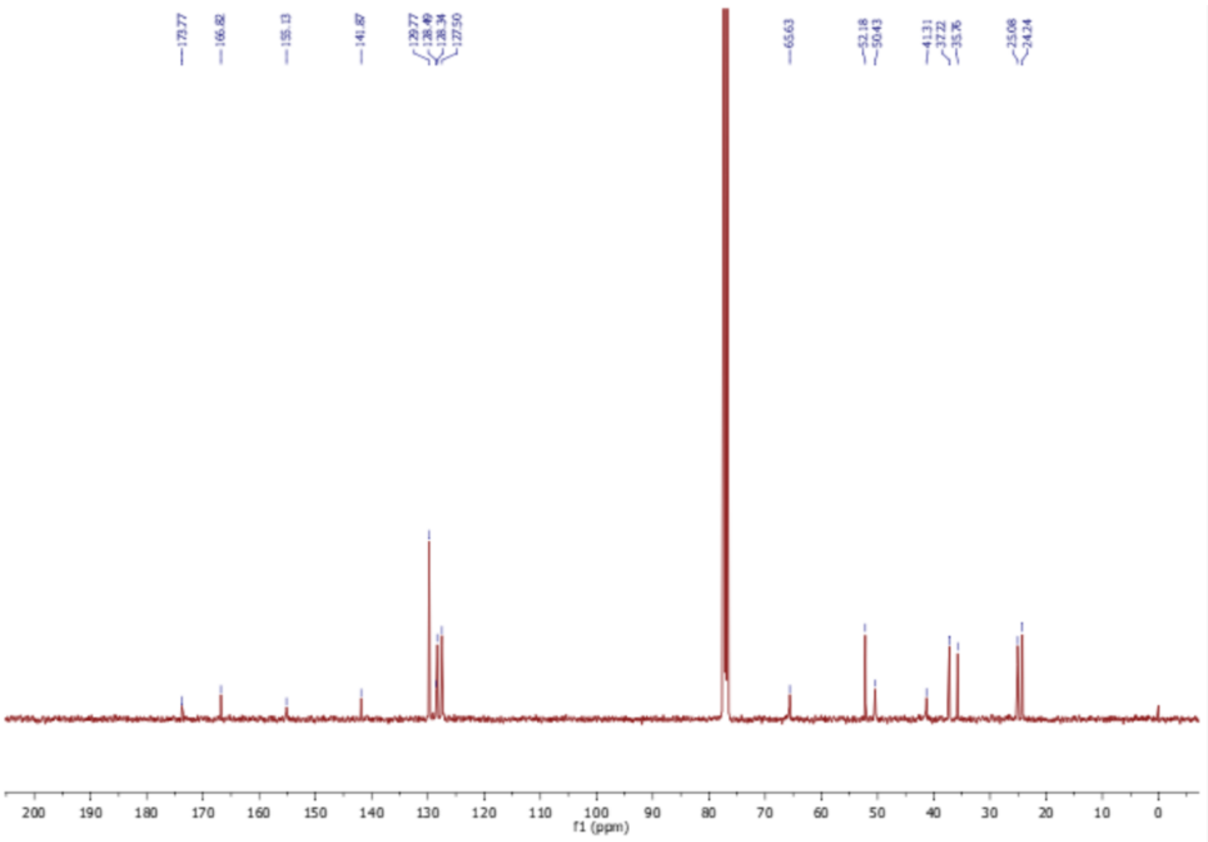

Supplementary Figure 15: ^{1}H NMR (300 MHz, $CDCl_3$) Spectrum of compound **3b**

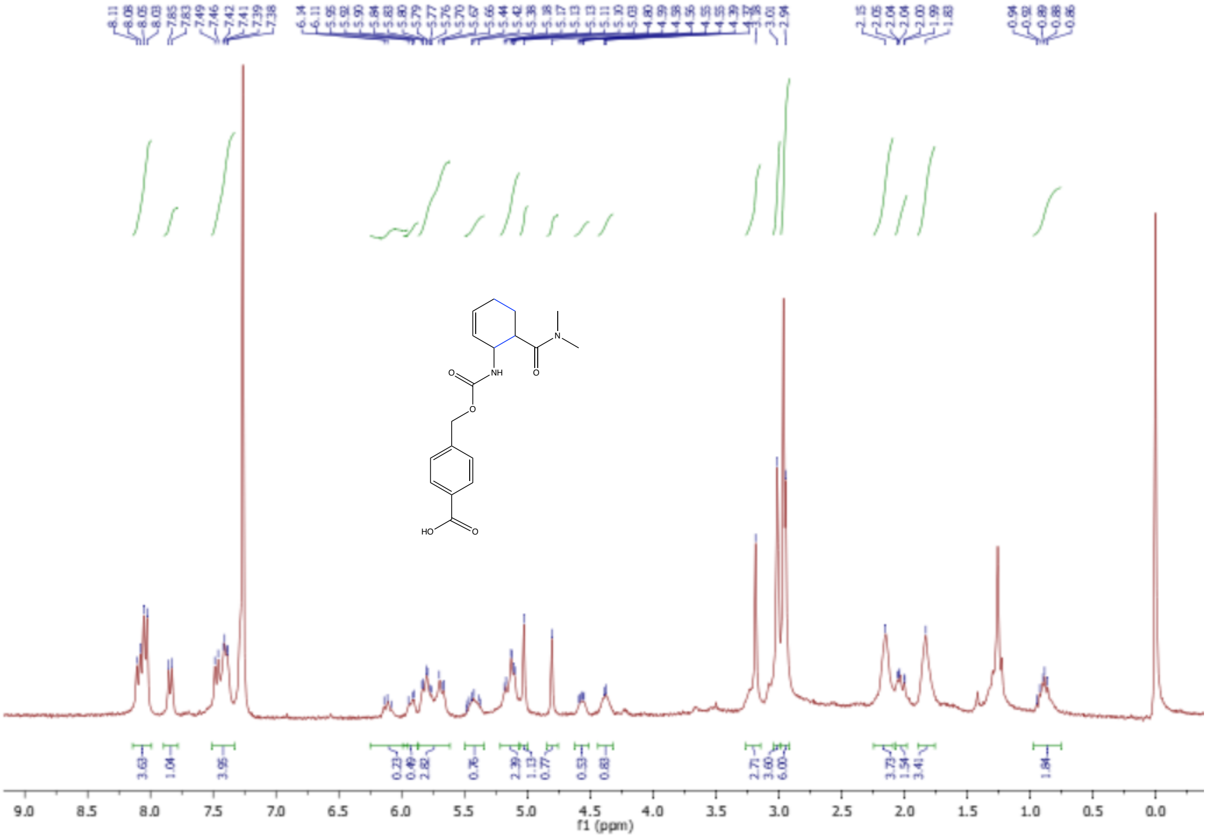

Supplementary Figure 16: a) ^{1}H NMR (400 MHz, $CDCl_3$) Spectrum of compound N, N-diethylacrylamide (**4**)

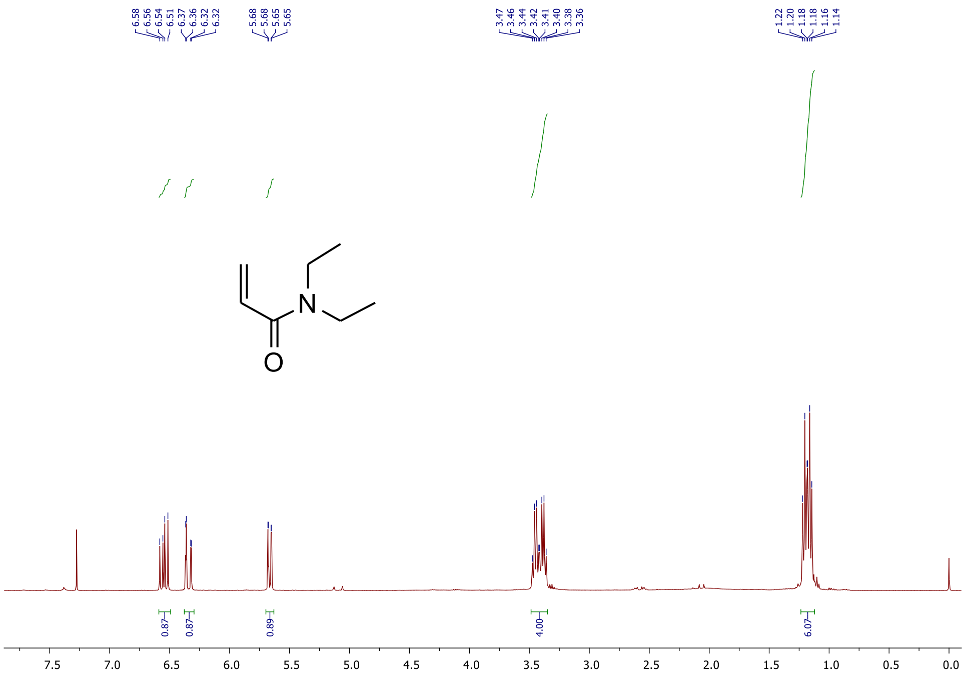

b) ^{13}C NMR (100 MHz, CDCl3) Spectrum of compound N,N-diethylacrylamide (**4**)

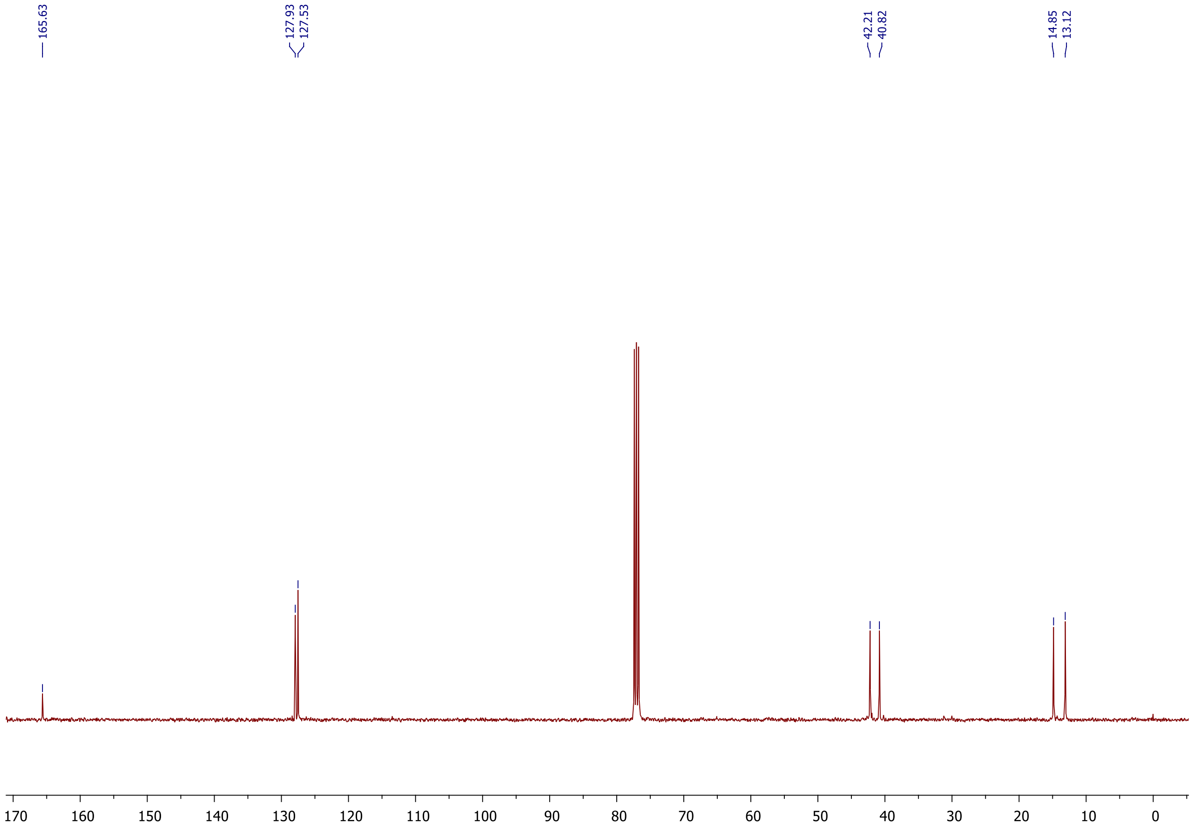

Supplementary Figure 17: a) ^{1}H NMR (400 MHz, $CDCl_3$) Spectrum of compound 1-morpholinoprop-2-en-1-one (**5**)

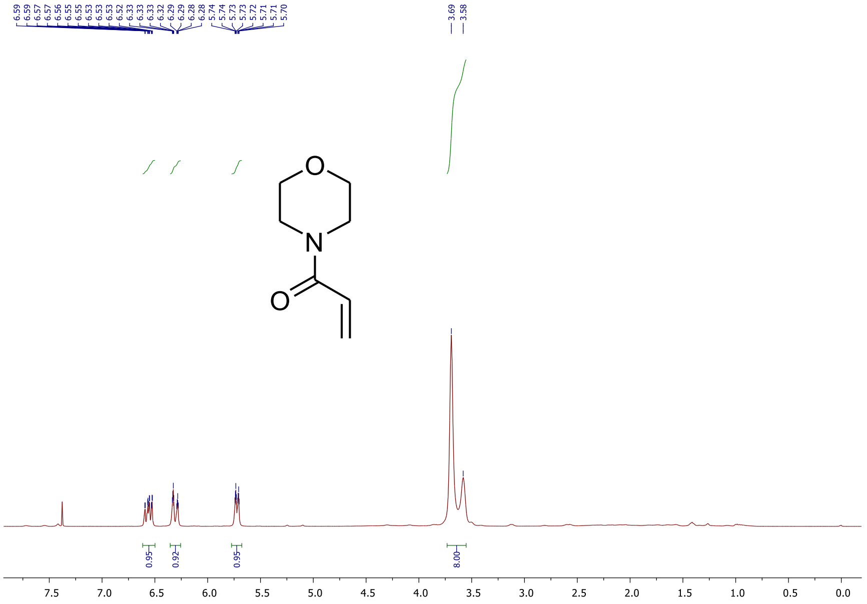

b) ^{13}C NMR (100 MHz, CDCl3) Spectrum of compound 1-morpholinoprop-2-en-1-one (**5**)

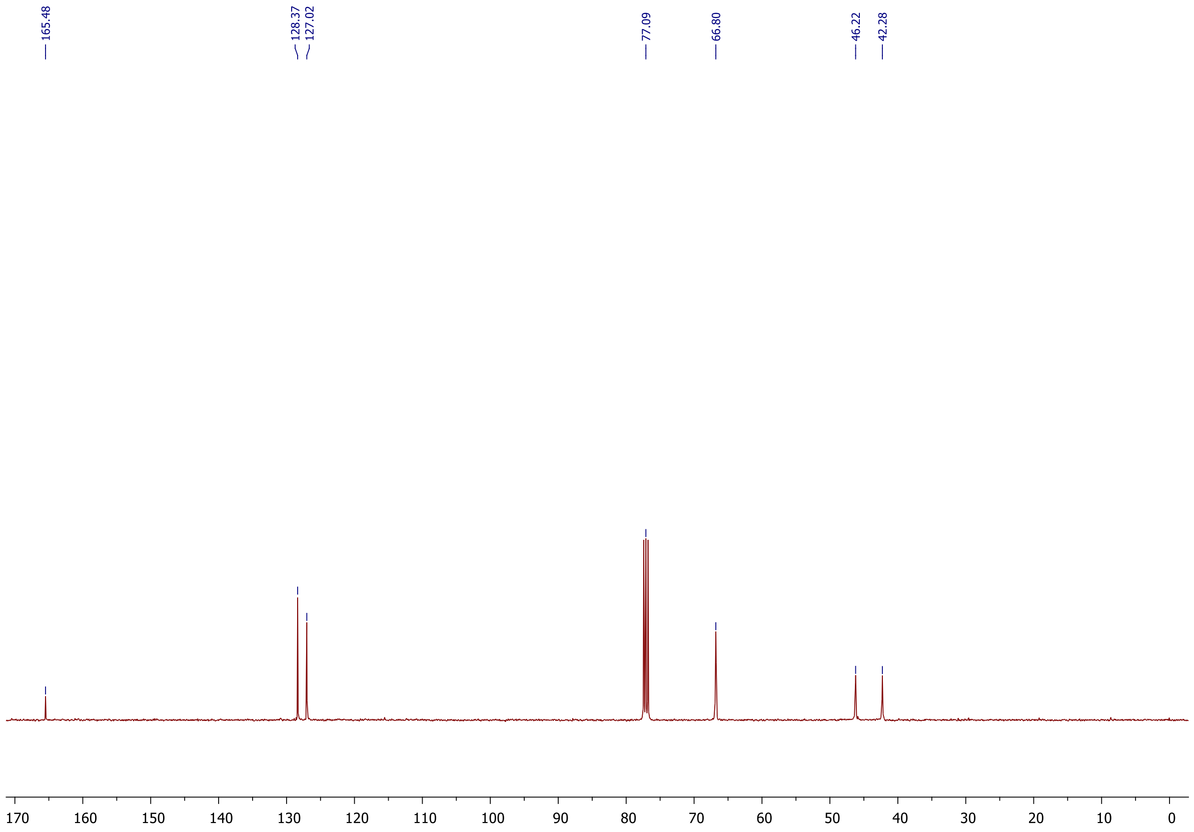

Supplementary Figure 18: a) ^{1}H NMR (400 MHz, $CDCl_3$) Spectrum of compound **6**

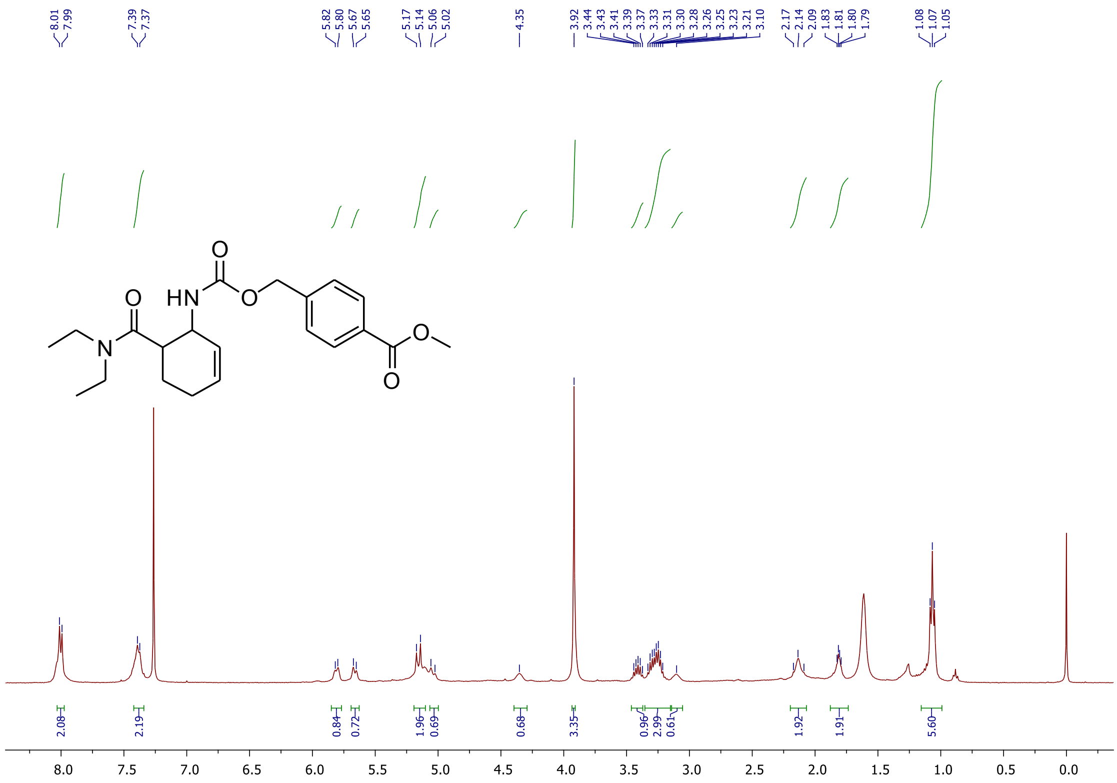

b) ^{13}C NMR (101 MHz, CDCl3) Spectrum of compound **6**

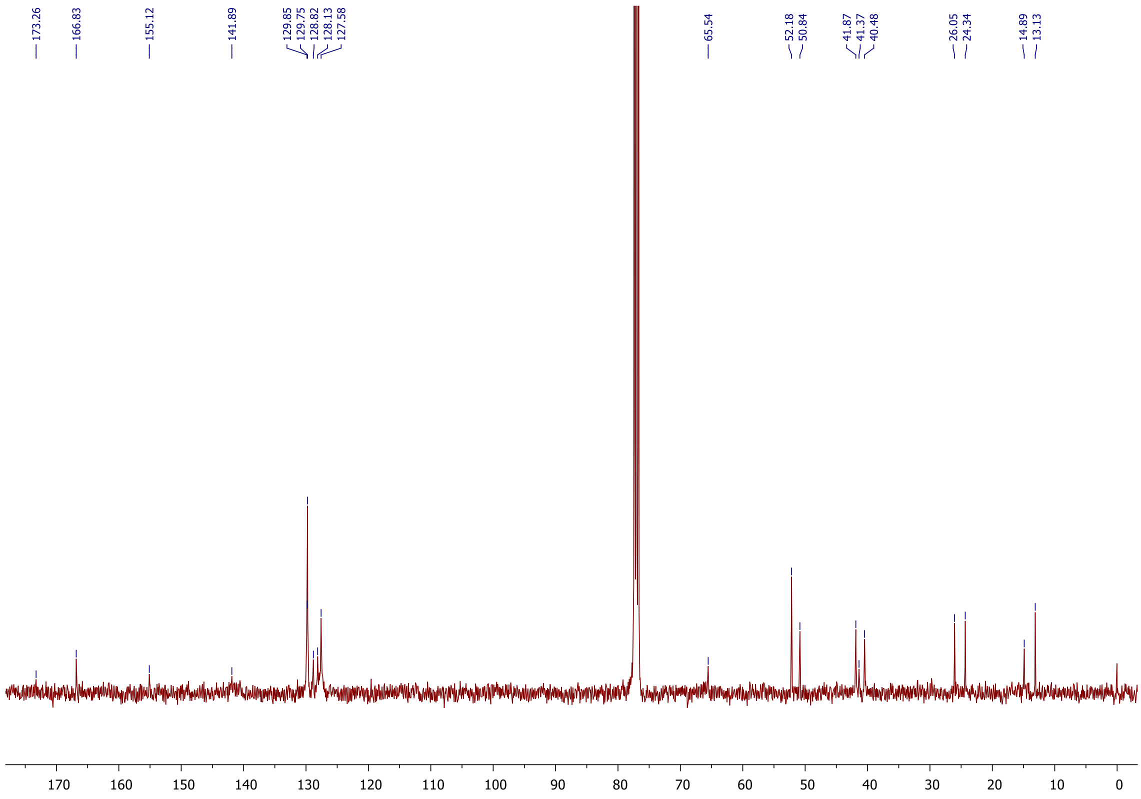

Supplementary Figure 19: a) ^{1}H NMR (400 MHz, CDCl3) Spectrum of compound **7**

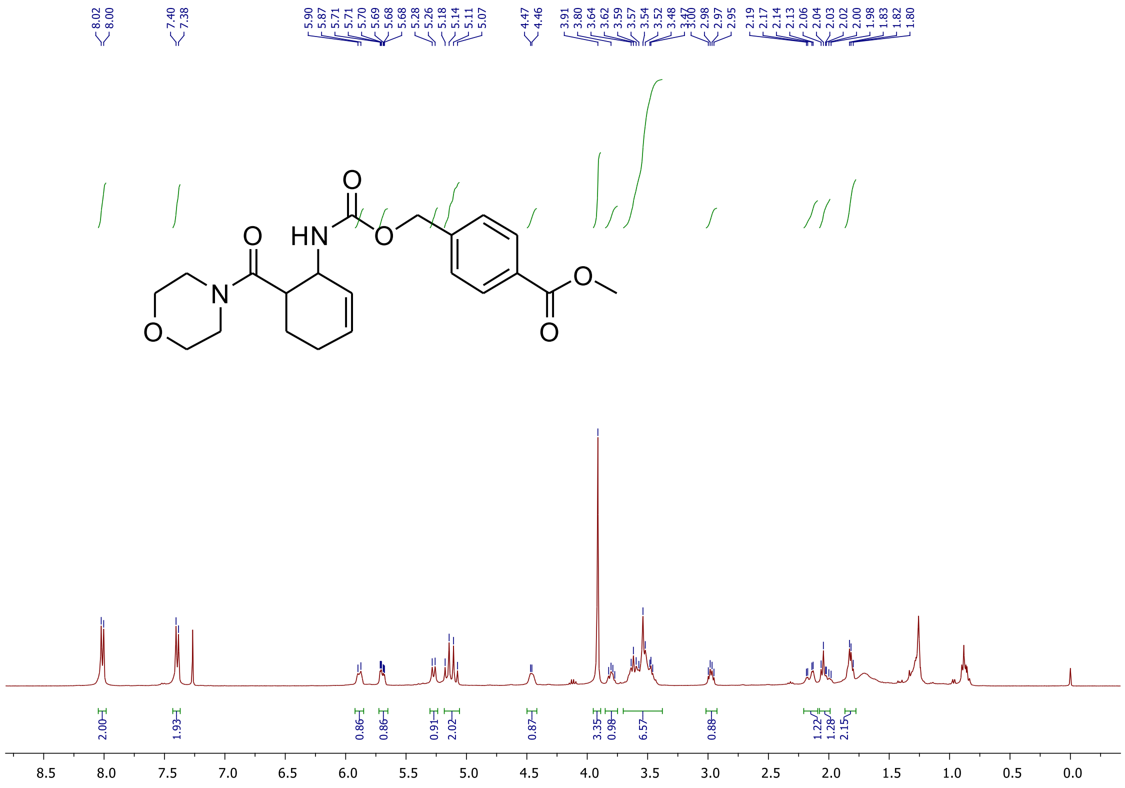

b) ^{13}C NMR (100 MHz, CDCl3) Spectrum of compound **7**

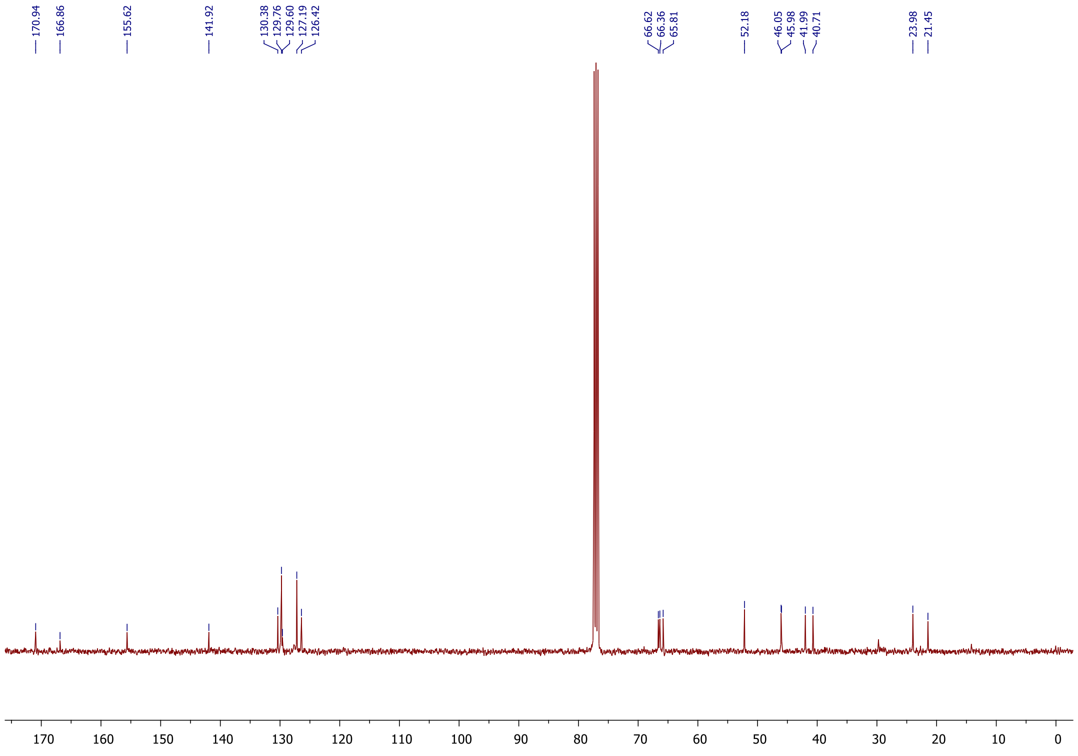

Supplementary References

1. Berman, H.M. et al. The Protein Data Bank. *Nucleic Acids Res* **28**, 235-242 (2000).
2. DeLano, W.L. The PyMOL Molecular Graphics System. *DeLano Scientific, San Carlos, CA, USA.* http://www.pymol.org., DeLano Scientific, San Carlos, CA, USA. http://www.pymol.org. (2002).
3. Waterhouse, A.M., Procter, J.B., Martin, D.M., Clamp, M. & Barton, G.J. Jalview Version 2--a multiple sequence alignment editor and analysis workbench. *Bioinformatics* **25**, 1189-1191 (2009).
4. Yerra, N.V. et al. 2-Cyano-3-(2-thienyl)acrylic Acid as a New MALDI Matrix for the Analysis of a Broad Spectrum of Analytes. *J Am Soc Mass Spectrom* **32**, 387-393 (2021).
5. Zheng, Q. et al. Enzyme-Dependent [4 + 2] Cycloaddition Depends on Lid-like Interaction of the N-Terminal Sequence with the Catalytic Core in PyrI4. *Cell Chem Biol* **23**, 352-360 (2016).
6. Siegel, J.B. et al. Computational design of an enzyme catalyst for a stereoselective bimolecular Diels-Alder reaction. *Science* **329**, 309-313 (2010).
7. Cannizzaro, C.E., Ashley, J.A., Janda, K.D. & Houk, K.N. Experimental determination of the absolute enantioselectivity of an antibody-catalyzed Diels-Alder reaction and theoretical explorations of the origins of stereoselectivity. *J Am Chem Soc* **125**, 2489-2506 (2003).
8. Gouverneur, V.E. et al. Control of the exo and endo pathways of the Diels-Alder reaction by antibody catalysis. *Science* **262**, 204-208 (1993).
9. Kabsch, W. Xds. *Acta Crystallogr D Biol Crystallogr* **66**, 125-132 (2010).
10. Evans, P.R. & Murshudov, G.N. How good are my data and what is the resolution? *Acta Crystallogr D Biol Crystallogr* **69**, 1204-1214 (2013).
11. Winn, M.D. et al. Overview of the CCP4 suite and current developments. *Acta Crystallogr D Biol Crystallogr* **67**, 235-242 (2011).
12. Vagin, A. & Teplyakov, A. Molecular replacement with MOLREP. *Acta Crystallogr D Biol Crystallogr* **66**, 22-25 (2010).
13. Emsley, P., Lohkamp, B., Scott, W.G. & Cowtan, K. Features and development of Coot. *Acta Crystallogr D Biol Crystallogr* **66**, 486-501 (2010).
14. Murshudov, G.N. et al. REFMAC5 for the refinement of macromolecular crystal structures. *Acta Crystallogr D Biol Crystallogr* **67**, 355-367 (2011).
